# Noncanonical Wnt5a signaling regulates tendon stem/progenitor cells senescence

**DOI:** 10.1186/s13287-021-02605-1

**Published:** 2021-10-18

**Authors:** Minhao Chen, Yingjuan Li, Longfei Xiao, Guangchun Dai, Panpan Lu, Yunfeng Rui

**Affiliations:** 1 Department of Orthopaedics, Zhongda Hospital, School of Medicine, Southeast University, No. 87 Ding Jia Qiao, Nanjing, 210009 Jiangsu People’s Republic of China; 2 Orthopaedic Trauma Institute (OTI), Southeast University, Nanjing, 210009 Jiangsu China; 3 Trauma Center, Zhongda Hospital, School of Medicine, Southeast University, Nanjing, 210009 Jiangsu China; 4 China Orthopedic Regenerative Medicine Group, Hangzhou, 310000 Zhejiang China; 5 Department of Geriatrics, Zhongda Hospital, School of Medicine, Southeast University, Nanjing, 210009 Jiangsu China

**Keywords:** Tendon-derived stem/progenitor cells, Senescence, Wnt5a, JAK–STAT, Ror2

## Abstract

**Background:**

The structural and functional properties of tendon decline with age, and these changes contribute to tendon disorder. Tendon stem/progenitor cells (TSPCs) play a vital role in tendon repair, regeneration and homeostasis maintaining. Although studies have demonstrated that tendon aging is closely associated with the altered TSPCs function on senescence, the cellular and molecular mechanisms of TSPCs senescence remain largely unknown. This study was designed to investigate the role of Wnt5a in TSPCs senescence.

**Methods:**

TSPCs were isolated from 2-month-old and 20-month-old male C57BL/6 mice. The expression of Wnt5a was determined by RNA sequencing, qRT-PCR and western blotting. TSPCs were then treated with Wnt5a shRNA or recombinant Wnt5a or AG490 or IFN-γ or Ror2-siRNA. Western blotting, β-gal staining, qRT-PCR, immunofluorescence staining and cell cycle analysis were used for confirming the role of Wnt5a in TSPCs senescence.

**Results:**

We found a canonical to noncanonical Wnt signaling shift due to enhanced expression of Wnt5a in aged TSPCs. Functionally, we demonstrated that inhibition of Wnt5a attenuated TSPCs senescence, age-related cell polarity and the senescence-associated secretory phenotype (SASP) expression in aged TSPCs. Mechanistically, the JAK–STAT signaling pathway was activated in aged TSPCs, while Wnt5a knockdown inhibited the JAK–STAT signaling pathway, suggesting that Wnt5a modulates TSPCs senescence via JAK–STAT signaling pathway. Moreover, knockdown of Ror2 inhibited Wnt5a-induced activation of the JAK–STAT signaling pathway, which indicates that Wnt5a potentiates JAK–STAT signaling pathway through Ror2, and Ror2 acts as the functional receptor of Wnt5a in TSPCs senescence.

**Conclusion:**

Our results demonstrate a critical role of noncanonical Wnt5a signaling in TSPCs senescence, and Wnt5a could be an attractive therapeutic target for antagonizing tendon aging.

**Supplementary Information:**

The online version contains supplementary material available at 10.1186/s13287-021-02605-1.

## Background

Tendon aging is an inevitable physiological process that results in impaired structural and functional properties of tendon, and these changes contribute to tendon disorder, such as chronic pain, tendon rapture and limited mobility [1, 2]. At the cellular level, tendon stem/progenitor cells (TSPCs), which exhibit various common properties of stem cells and have been isolated from various species [3–5], undergo senescence along with tendon aging [6]. TSPCs express typical tendon-lineage genes and exhibit clonogenicity, self-renewal and multiple differentiation capacity. TSPCs play a critical role in tendon repair, regeneration and homeostasis maintaining [7–9]. Studies have shown that TSPCs senescence is closely associated with the age-related tendon disorder. As compared with young cells, aged TSPCs display deficient self-renewal, migration and tenogenic differentiation capacity, as well as substantial changes in transcriptome, leading to impaired tendon healing and regeneration capacity [10, 11]. Although the decline of TSPCs function on senescence has been well recognized, the molecular mechanisms of this process are still largely unknown.

The Wnt signaling plays critical roles in growth and development of multiple tissues, including musculoskeletal system, hematopoietic system and digestive system [12–14]. Wnt family proteins exert two signaling pathways based on their dependence on transduction through β-catenin: the canonical Wnt pathway and the noncanonical Wnt pathway [15, 16]. Noncanonical Wnt pathway can inhibit canonical Wnt pathway, causing attenuated β-catenin stability and impaired downstream pathway [17, 18]. Wnt5a is a prototypical ligand for the noncanonical Wnt pathway and has been reported to play a critical role in aging. In aged lung, increased Wnt5a stimulates inflammation and maintenance of a damaging environment to the gas-exchange surface [19]. Jung et al. demonstrated that Wnt5a is a downstream effector of UHRF1/DNMT1-mediated senescence in human diploid fibroblasts (HDFs). Wnt5a overexpression in young HDFs also induced senescent phenotypes [20]. In hematopoietic stem cells (HSCs), Wnt5a expression was observed to increase with age, which causes HSCs senescence. Wnt5a haploinsufficiency attenuates HSCs senescence, whereas Wnt5a knockdown results in functionally rejuvenated aged HSCs [21]. In addition, a few studies have indicated that Wnt5a is also involved in the regulation of tendon stem cells [22, 23]. Yu et al. reported that uniaxial mechanical tension promoted the osteogenic differentiation of TSPCs through the Wnt5a-RhoA pathway [23]. Although studies have suggested the role of Wnt5a in aging and regulation of tendon stem cells, there were no studies focused on the role of Wnt5a in TSPCs senescence during tendon aging.

Here, we showed a switch from canonical to noncanonical Wnt signaling pathway due to elevated expression of Wnt5a in aged TSPCs. We confirmed the critical role of Wnt5a in TSPCs senescence, and Wnt5a knockdown could attenuate TSPCs senescence. Wnt5a regulates TSPCs senescence through JAK–STAT signaling pathway. In addition, we found that Ror2 acts as the functional receptor of Wnt5a in TSPCs senescence. Our results provide evidence of noncanonical Wnt5a signaling WNT signaling being crucially involved in TSPCs senescence.

## Materials and methods

### TSPCs isolation and culture

Isolation of TSPCs from achilles tendon was performed as previously described [4]. Briefly, mouse achilles tendons were isolated from 2-month-old (abbreviated as Y-TSPC) and 20-month-old (abbreviated as A-TSPC) male C57BL/6 mice (n = 20). After gently mincing the achilles tendons, the chopped tissue was digested with 3 mg/mL collagenase type I (Sigma–Aldrich) and 4 mg/mL dispase (Roche), subsequently percolating with a 70-μm cell strainer (Becton Dickinson) to obtain a single-cell suspension. The released cells were washed in phosphate-buffered saline (PBS) and resuspended in alpha-minimum essential medium (α-MEM) containing 20% fetal bovine serum (Gibco), 2 mM L-glutamine (Gibco), 100 μM 2-mercaptoethanol (Sigma–Aldrich) and 1% penicillin–streptomycin (Gibco). An optimal low cell density (50 nucleated cells/cm^2^) was applied to plant the isolated nucleated cells for forming colonies at 37 °C, 5% CO_2_. At day 7, cell colonies were trypsinized and mixed together as passage 0 (P0). Cells from P2 to P6 were used for all experiments. Medium was changed every 3 days. All surgical interventions and postoperative animal care were performed according to the Guide for the Care and Use of Laboratory Animals (National Research Council) and were approved by the Animal Research Ethics Committee of Southeast University. Experimental design aimed to minimize the animal number and their sacrifice.

### Cell transfection

Lentivirus-mediated Wnt5a short-hairpin RNA labeled with GFP component (Wnt5a shRNA) and negative control lentivirus (LV-GFP) were acquired from GeneChem Corporation (Shanghai, China), and the target sequence of Wnt5a shRNA was as follows: gtGGATCAGTTCGTGTGCAAA. 8 × 10^4^ cells were plated on 6-well plates and cultured to reach 20–30% confluence before transfection. Lentivirus was transfected into cells by HitransG Transfection Reagent P (Genechem) for 16 h, and then, the transfected cells were selected with 2 μg/ml puromycin (Beyotime Biotechnology) for 10 days to establish stably expressing cells and verified by western blotting.

Ror2-siRNA was acquired from GenePharma (Shanghai, China), and the sequence of Ror2-siRNA was as follows: 5'-GGUUUGGCAAGGUCUACAATT-3'. 1 × 10^5^ Cells were plated on 6-well plates and transfected at 50% confluence. The jetPRIME transfection reagent (Polyplus) was mixed with Ror2-siRNA and incubated for 10 min at room temperature. Then, the mixture was added to the cells in serum-containing medium and incubated for 48–72 h before cells were harvested for subsequent experiments.

### RNA sequencing

The profiles of gene expression were tested in Beijing CapitalBio Corporation (Beijing, China). In brief, with the usage of poly-T oligo-attached magnetic beads, the purified poly(A)-containing mRNA molecules were extracted from total RNA (3 μg). The cleaved RNA fragments were reversely transcribed to first-strand cDNA utilizing random hexamers and then to second-strand cDNA synthesis with DNA polymerase I and RNase H. The cDNA fragments were purified with end blunted, 'A' tailed and adaptor ligated. Afterward, PCR was used to selectively enrich those DNA fragments with adapter molecules on both ends, in the purpose of amplify the amount of DNA in the library which was certified by Agilent 2100 Bioanalyzer and quantified by Qubit and qPCR. Meanwhile, the representation of the library from skewing with a minimized number of PCR cycles was prevented and the produced libraries were sequenced with HiSeq 2500 platform. With robust multi-array average normalization, fold change threshold ≥ 2 and P value < 0.05 can be viewed as statistically significant alterations. With Cluster3.0 software, clustering analysis and heatmap generation were executed. The functional assignments were mapped onto gene ontology (GO). Gene set enrichment analysis (GSEA, http://software.broadinstitute.org/gsea/index.jsp) was performed to verify the biological processes in the two groups as mentioned above [24]. The calculation of normalized enrichment score (NES) and false discovery rate (FDR) was used to verify the significant difference for GSEA.

### Western blotting

To detect the expression of target proteins, western blotting was performed. Total cell protein was extracted in cold RIPA lysis buffer (Keygen Biotech) after being cultured with or without Wnt5a shRNA/Ror2-siRNA/recombinant Wnt5a (R&D System)/IFN-γ (Beyotime Biotechnology)/AG490 (MCE). Protein concentration was measured using the BCA protein assay kit (Thermo Scientific). A total of 30 μg of protein was electrophoresed on SDS–PAGE and then electrotransferred to a PVDF membrane (Millipore). Nonspecific binding was blocked with PBST containing 5% nonfat dry milk, followed by incubation with primary antibody against Wnt5a (Cell Signaling Technology), β-catenin (Abcam), p16^INK4A^ (Abcam), JAK2 (Proteintech), p-JAK2 (Abcam), STAT3 (Proteintech), p-STAT3 (Abcam) and GAPDH (Proteintech) at 4 °C overnight. Immunoreactive bands were incubated with secondary antibody, before detecting it with ECL reagents (Keygen biotech). The gray value of each band was measured, data of which were presented as a ratio to GAPDH.

### Immunofluorescence staining

To examine localization of target proteins, cultured TSPCs were fixed in 4% paraformaldehyde for 15 min at room temperature. Pretreatment with 0.2% Triton X-100 in PBS for 20 min, cells were blocked with 3% bovine serum albumin for 30 min at room temperature. After being washed, cells were incubated overnight at 4 °C with anti-Wnt5a (Santa Cruz), β-catenin (Abcam) and α-tubulin (Abcam), followed by a mixture of AlexaFluor 594-conjugated secondary antibodies (Molecular Probes) incubated for 2 h at room temperature. Immunofluorescence images were captured with a Nikon Ts2R fluorescence microscope.

### β-galactosidase staining

Cellular senescence assays were performed using the β-galactosidase (β-gal) staining kit (Sigma) according to the manufacturer's protocol. Briefly, cells were plated on 12-well plates and cultured for 48 h, and then, cells were incubated with the kit’s staining mixture for 16 h at 37 °C. The percentages of β-gal-positive cells were then calculated in six microscopic fields. Images were captured with an Olympus CKX53 inverted phase-contrast microscope.

### Quantitative RT-PCR

To examine mRNA level of target genes, total RNA was purified from TSPCs by using the MiniBEST universal RNA extraction kit (Takara). The mRNA was reversely transcribed to cDNA with the first-strand cDNA kit (Promega). With the utilization of the ABI Step One Plus system (Applied Biosystems), each sample with 1 μL total cDNA was amplified in the final volume of 20 μL of reaction mixture containing Power SYBR Green PCR Master Mix (Invitrogen) and specific primers. Denaturation was performed at 95 °C for 10 min, 45 cycles at 95 °C for 20 s, optimal annealing temperature for 20 s or 72 °C for 30 s and finally at 60 °C–95°C with a heating rate of 0.1 °C/s. Genes were normalized to that of the β-actin gene. Relative gene expression was calculated using 2^−ΔΔCt^ method. Additional file 3: Table S2 shows primer sequences used in this study.

### Cell cycle analysis

To detect cell cycle distribution, TSPCs were cultured on 10-cm dishes in 2% FBS/DMEM for 48 h, and then, cells were trypsinized, detached and fixed in 70% ethanol overnight at 4 °C. Subsequently, the ethanol-fixed cells were incubated with RNase (Keygen biotech) and propidium iodine (Keygen biotech) for 30 min. Using flow cytometry (Becton Dickinson) with Cell Quest software, the percentages of cells in the three phases of the growth cycle (G1, S and G2/M phase) were measured.

### Statistical analysis

Statistical analysis was performed with SPSS version 16.0 (SPSS). All data were plotted as mean ± SEM. Two groups were compared with unpaired t-test. One-way analysis of variance (ANOVA) and Tukey’s post hoc test were used for multiple comparisons, with *p* < 0.05 considered statistically significant. In addition, there are at least three replicates per condition made up of each experiment. Groups with similar variance were being statistically compared.

## Results

### Wnt5a mediates a switch from canonical to noncanonical Wnt signaling in aged TSPCs

**Fig. 1:**
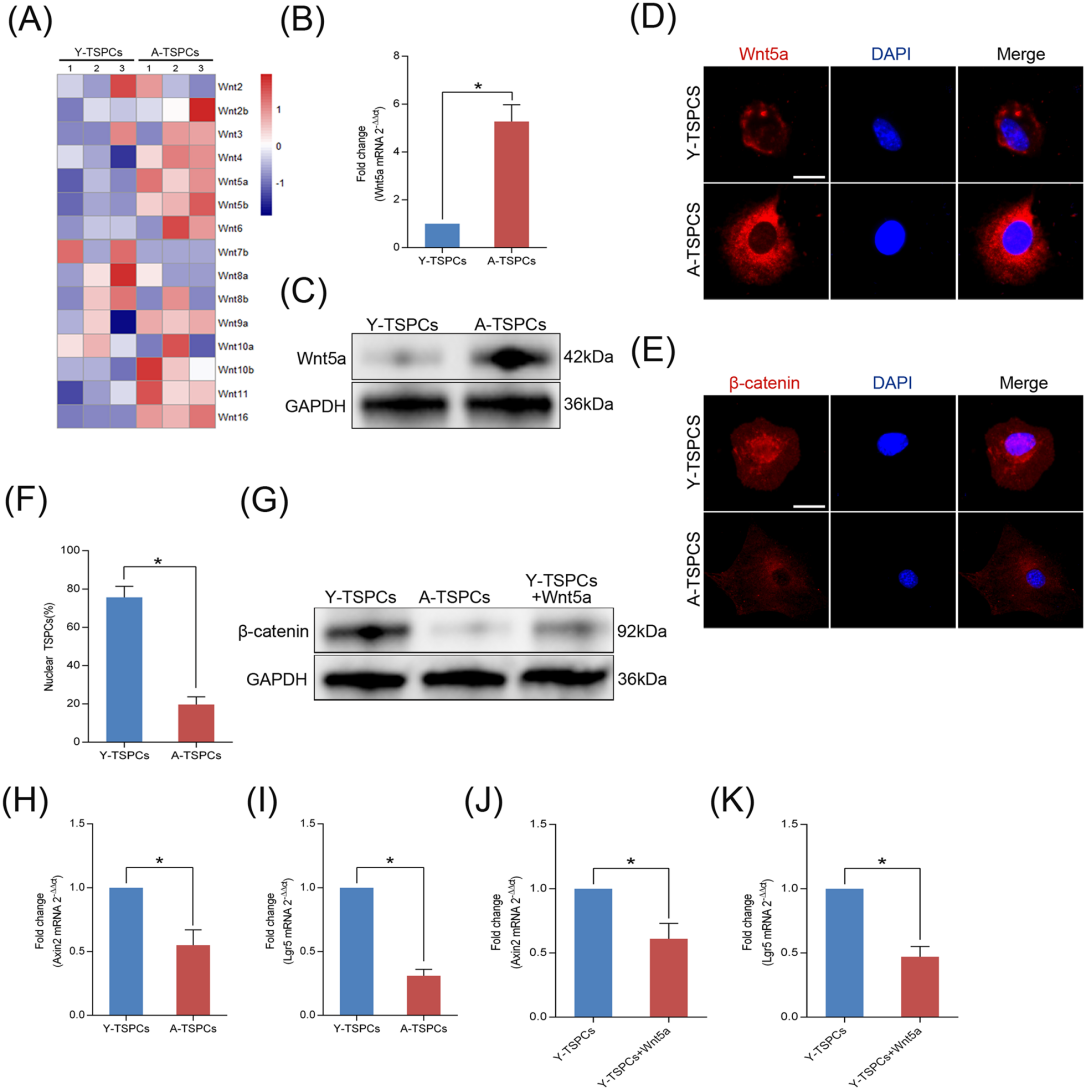
Increased Wnt5a expression correlates with a switch from canonical to noncanonical Wnt signaling. **a** Heatmap showed the changes in the expression of Wnt genes in young and aged TSPCs. **b** Relative mRNA levels of Wnt5a in TSPCs were investigated by qRT-PCR. **c** Western blotting for the Wnt5a protein levels in young and aged TSPCs. **d** Immunofluorescence staining for Wnt5a in young and aged TSPCs. Scale bar: 20 μm. **e**, **f** Immunofluorescence staining for β-catenin in young and aged TSPCs. Scale bar: 20 μm. **g** Western blotting for β-catenin protein levels in young TSPCs, aged TSPCs and young TSPCs treated with Wnt5a (100 ng/ml). **h**, **i** qRT-PCR for Axin2 and Lgr5 mRNA levels in young and aged TSPCs. **j**, **k** Axin2 and Lgr5 mRNA levels in young TSPCs and young TSPCs treated with Wnt5a. Values represent the mean ± SD. *p < 0.05, significantly different from the young group

TSPCs were isolated from young (2-month-old) and aged (20-month-old) mouse achilles tendon (Additional file 5: Fig. S1) and characterized in vitro (Additional file 6: Fig. S2). We first examined the mRNA expression profiles by RNA-seq analysis on young and aged TSPCs (Additional file 7: Fig. S3), we then analyzed Wnt family microarray data and found a marked increase in the expression of Wnt5a (Fig. 1a), while other members of the canonical Wnt family (such as Wnt2, Wnt2b, Wnt3, Wnt10a and Wnt10b) did not present with significant changes in expression on TSPCs senescence. We confirmed the results by qRT-PCR to examine expression of Wnt5a gene (Fig. 1b). Consistent with the qRT-PCR results, Wnt5a protein level was also markedly increased in aged TSPCs (Fig. 1c, d). In addition, immunofluorescence staining revealed that β-catenin was expressed in the nucleus in young TSPCs, which suggested the activation of canonical Wnt signaling in young TSPCs (Fig. 1e, f). In aged TSPCs, β-catenin was predominantly located in cytoplasm (Fig. 1e, f). We also found that the protein level of β-catenin is reduced in aged TSPCs, and recombinant Wnt5a treatment reduced the expression of β-catenin in young TSPCs, indicating a direct action of Wnt5a on β-catenin expression in TSPCs (Fig. 1g). We next investigated the direct downstream target genes of canonical Wnt signaling, Axin2 and Lgr5, which were also significantly reduced in aged TSPCs (Fig. 1h, i). Moreover, the expressions of Axin2 and Lgr5 were decreased in young TSPCs after recombinant Wnt5a treatment (Fig. 1j, k). The results suggested that TSPCs senescence is accompanied by a shift from canonical to noncanonical Wnt signaling due to elevated Wnt5a expression.

### Wnt5a plays a critical role in TSPCs senescence

**Fig. 2:**
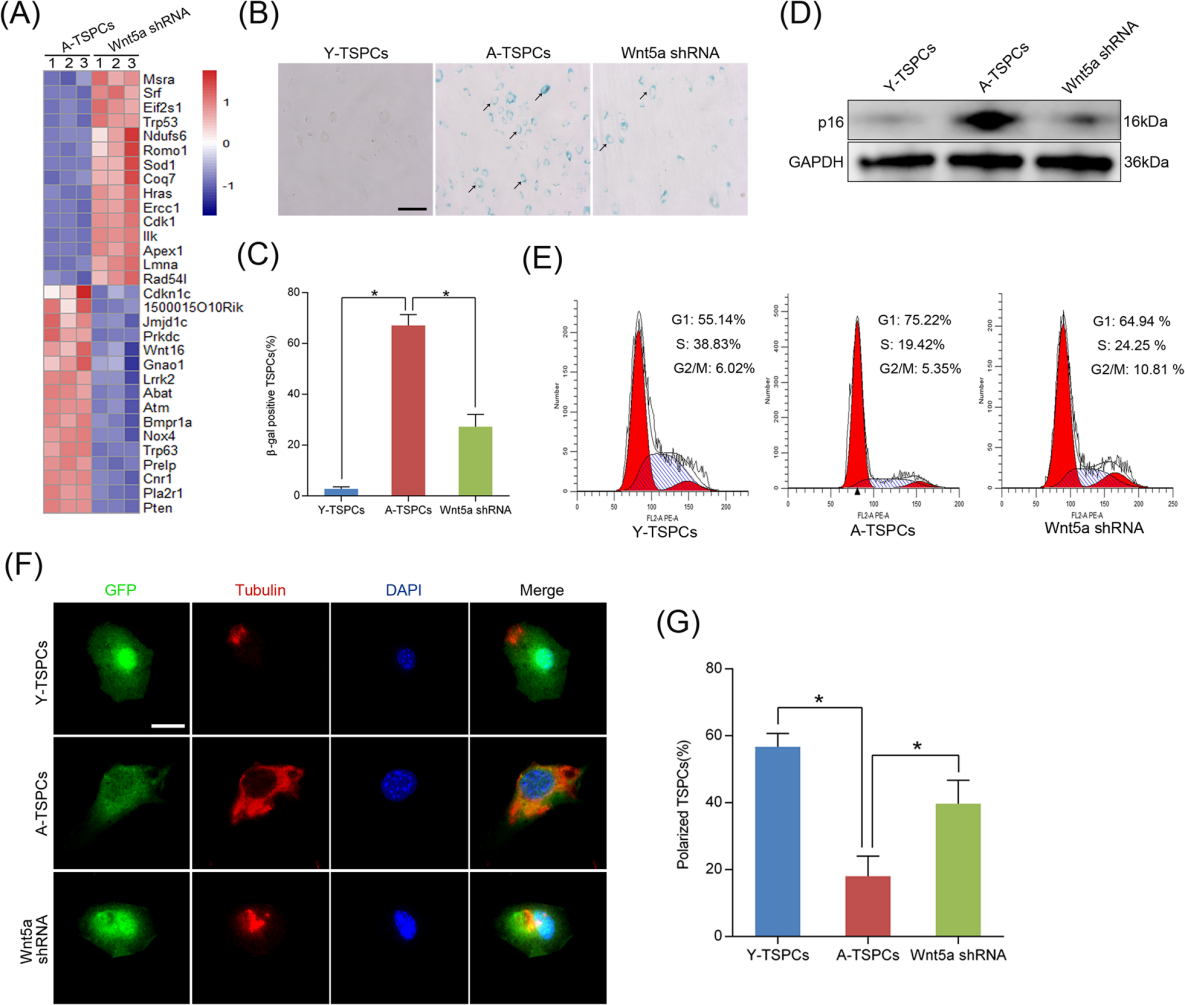
Wnt5a knockdown attenuates TSPCs senescence. **a** Heatmap of aging-related genes expressions in aged and Wnt5a-knockdown aged TSPCs. **b**, **c** β-gal staining for the senescent cells in young, aged and Wnt5a-knockdown aged TSPCs. Scale bars: 100 μm. **d** Western blotting for p16^INK4A^ protein levels in young, aged and aged Wnt5a-knockdown TSPCs. **e** Flow cytometry analysis was used to measure the cell cycle distribution of TSPCs. **f**, **g** Representative distribution of tubulin in young, aged and aged Wnt5a-knockdown TSPCs was investigated by immunofluorescence. Scale bar: 20 μm. Values represent the mean ± SD. *p < 0.05, significantly different from the young or aged group

To investigate the role of Wnt5a in TSPCs senescence, we knockdown the cell-intrinsic Wnt5a through lentiviral short-hairpin RNA (Wnt5a shRNA) in aged TSPCs. Of note, RNA-seq analysis showed remarkable differences between aged TSPCs and Wnt5a-knockdown aged TSPCs (Additional file 8: Fig. S4). We further found a marked change in the expression of aging-related genes, including genes involved in tendon aging, such as Cdkn1c and Trp53 (Fig. 2a). In addition, silencing Wnt5a significantly reduced β-gal-positive senescence cells in aged TSPCs (Fig. 2b, c), and this was also coupled with a significant repression of senescence marker p16^INK4A^ in aged TSPCs (Fig. 2d). Senescence represents a state of permanent cell cycle arrest; here, we investigated cell cycle phase distribution of TSPCs, aged TSPCs exhibited an increased proportion in the G1 phase, while the accumulation of aged TSPCs at G1 phase was blocked after Wnt5a shRNA treatment (Fig. 2e). Studies have demonstrated that age-related dysfunction of several stem cell populations correlates with altered cell polarity [25, 26]. In the present study, we used tubulin as a marker for cell polarization, and we found that tubulin was asymmetrically distributed in young TSPCs, while tubulin was distributed throughout the cell body in aged TSPCs, which suggested a unpolarized phenotype of aged TSPCs (Fig. 2f, g). Notably, Wnt5a shRNA treatment significantly increased the frequency of polarized cells in aged TSPCs. To further investigate the role of Wnt5a in TSPCs senescence, we treat young TSPCs with recombinant Wnt5a. The results showed that Wnt5a increased the expression of p16^INK4A^, as well as the β-gal-positive senescence cells in young TSPCs. Moreover, Wnt5a treatment also induced the accumulation of young TSPCs in G1 phase (Additional file 9: Fig. S5). Collectively, the data showed the critical role of Wnt5a in TSPCs senescence.

### Wnt5a knockdown inhibits the SASP expression in aged TSPCs

**Fig. 3:**
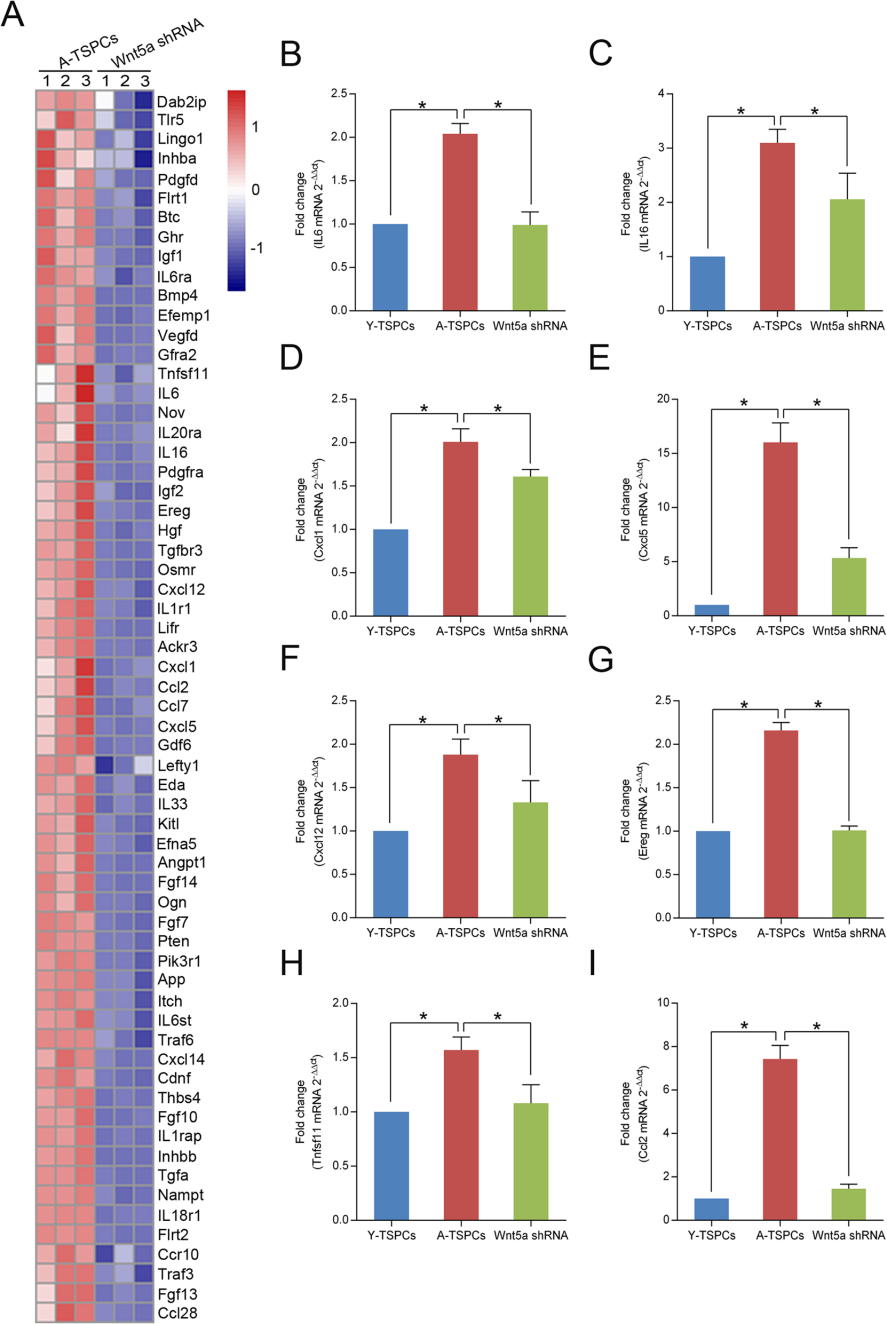
Effect of Wnt5a shRNA on SASP expression in aged TSPCs. **a** Heatmap of representative SASP genes expressions identified by a combination of GO analysis in aged and Wnt5a-knockdown aged TSPCs. **b**–**i** Relative mRNA levels of representative SASP genes (IL6, IL16, Cxcl1, Cxcl5, Cxcl12, Ereg, Tnfsf11, Ccl2) in young, aged and aged Wnt5a-knockdown TSPCs were investigated by qRT-PCR. Values represent the mean ± SD. *p < 0.05, significantly different from the young or aged group

Given the ability of Wnt5a shRNA to attenuate TSPCs senescence, we next investigated whether Wnt5a knockdown affects the senescence-associated secretory phenotype (SASP) expression of senescent TSPCs. Using RNA-seq, we found a marked change in the expression of genes involved in numerous categories associated with the SASP by gene ontology (GO) analysis (including cytokine activity, cytokine receptor activity, cytokine receptor binding, chemokine activity, growth factor activity and growth factor receptor binding) (Additional file 2: Table S1). We then identified the down-regulated genes from aged TSPCs to Wnt5a-knockdown aged TSPCs (Fig. 3a). We confirmed the results by qRT-PCR to examine expression of several representative SASP genes, including IL6, IL16, Cxcl1, Cxcl5, Cxcl12, Ereg, Tnfsf11 and Ccl2 (Fig. 3b–i). Compared to young TSPCs, these SASP genes were significantly increased in aged TSPCs, and Wnt5a shRNA treatment could rescue the increased level of the SASP genes. Together, the results confirmed the ability of Wnt5a knockdown attenuates SASP expression.

### Wnt5a promotes TSPCs senescence through JAK–STAT signaling pathway

**Fig. 4:**
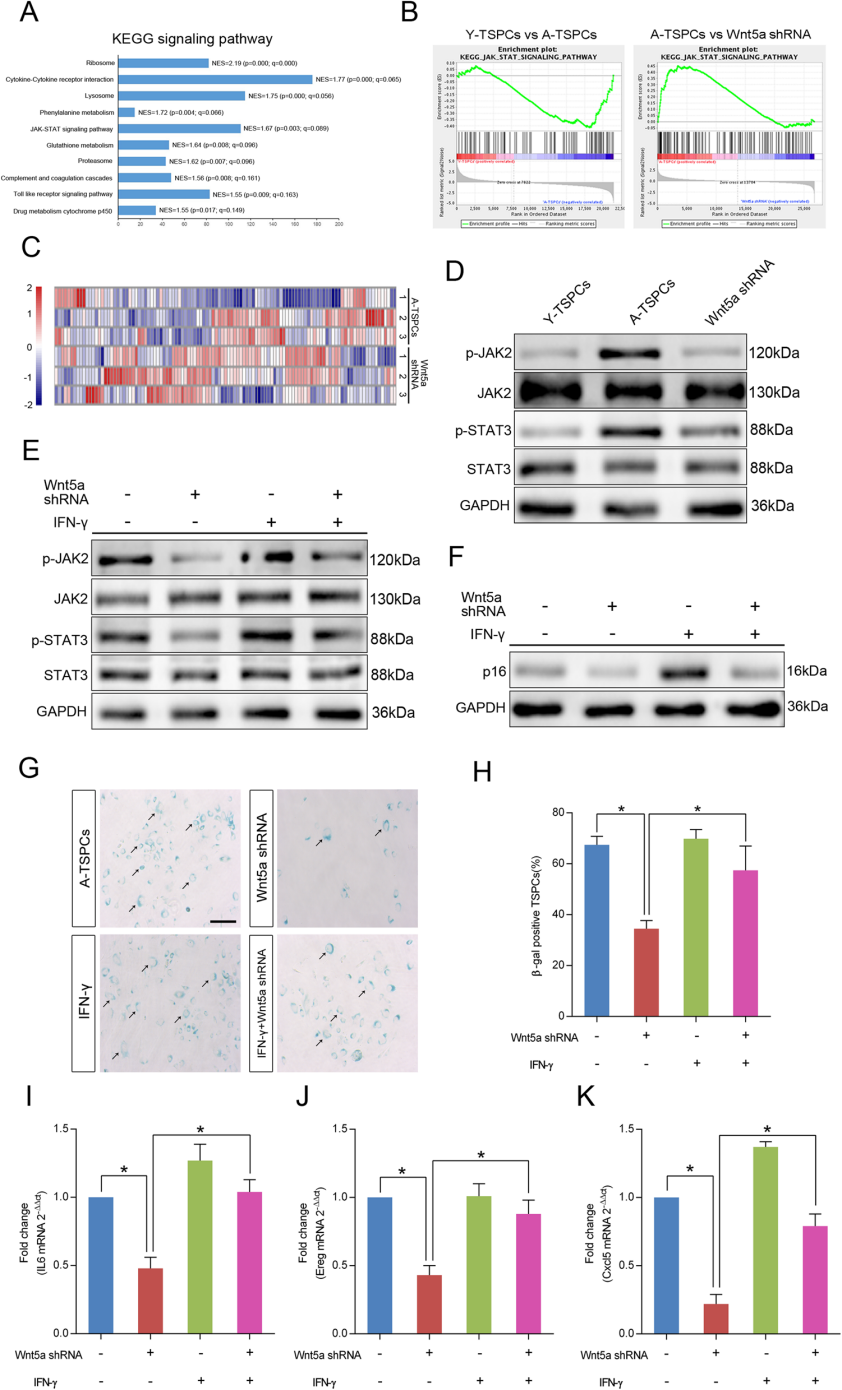
Wnt5a activates JAK–STAT signaling pathway in TSPCs senescence. **a** The GSEA KEGG analysis revealed the top 10 significantly enriched signaling pathways in Wnt5a-knockdown aged TSPCs. **b** GSEA plots showed a gene set related to JAK–STAT pathway. **c** Heatmap of relative expression of the genes involved in the JAK–STAT signaling pathways in aged and Wnt5a-knockdown aged TSPCs. **d** Western blotting for the p-JAK2, JAK2, p-STAT3 and STAT3 protein levels in young, aged and Wnt5a-knockdown aged TSPCs. **e** Aged TSPCs were transfected with or without Wnt5a shRNA and then stimulated with IFN-γ at 100 ng/ml for 8 h. The protein levels of p-JAK2, JAK2, p-STAT3 and STAT3 were investigated by western blotting. **f** Western blotting for the p16^INK4A^ protein levels in aged TSPCs upon Wnt5a shRNA and/or IFN-γ treatment for 8 h. **g**, **h** β-gal staining in aged TSPCs upon Wnt5a shRNA and/or IFN-γ (100 ng/ml, 24 h) treatment. Scale bars: 100 μm. **i**–**k** Relative mRNA levels of SASP genes (IL6, Ereg and Cxcl5) in aged TSPCs upon Wnt5a shRNA and/or IFN-γ (100 ng/ml, 8 h) treatment. Values represent the mean ± SD. *p < 0.05, significantly different from the young or aged group

To investigate critical signaling pathways involved in the TSPCs senescence regulated by Wnt5a, we used gene set enrichment analysis (GSEA) to identify enriched signaling pathways in aged and Wnt5a-knockdown aged TSPCs (Fig. 4a and Additional file 10: Fig. S6). Notably, JAK–STAT signaling pathway has been reported to be linked to cellular senescence in several cell types [27, 28], and GSEA showed that gene set related to JAK–STAT signaling pathway was reduced in Wnt5a-knockdown aged TSPCs (Fig. 4b). The heatmap showed the gene set involved in JAK–STAT signaling pathway (Fig. 4c). We then validated the expressions of genes involved in JAK–STAT signaling pathway by western blotting, and the result showed that the phosphorylation levels of JAK2 and STAT3 were significantly increased in aged TSPCs, which suggested the activation of JAK–STAT signaling, and Wnt5a knockdown reduced the expressions of p-JAK2 and p-STAT3 (Fig. 4d). In contrast, Wnt5a treatment increased the expressions of p-JAK2 and p-STAT3 in young TSPCs (Additional file 11: Fig. S7). We next stimulated aged TSPCs with interferon-γ (IFN-γ), an activator of JAK–STAT signaling pathway [29], and the results showed that IFN-γ treatment abolished the inhibition effects of Wnt5a shRNA on JAK–STAT signaling pathway and TSPCs senescence hallmarks, including reduced p16^INK4A^ protein level (Fig. 4f), number of β-gal-positive senescent cells (Fig. 4g, h) and SASP genes expressions (Fig. 6i–k). In addition, we treated young TSPCs with JAK–STAT signaling pathway inhibitor AG490; of note, AG490 treatment also reversed the Wnt5a-induced senescence of TSPCs (Additional file 11: Fig. S7). Together, the results indicate that Wnt5a regulates TSPCs senescence through JAK–STAT signaling pathway during tendon aging.

### Ror2 acts as the functional receptor of Wnt5a in TSPCs senescence

**Fig. 5:**
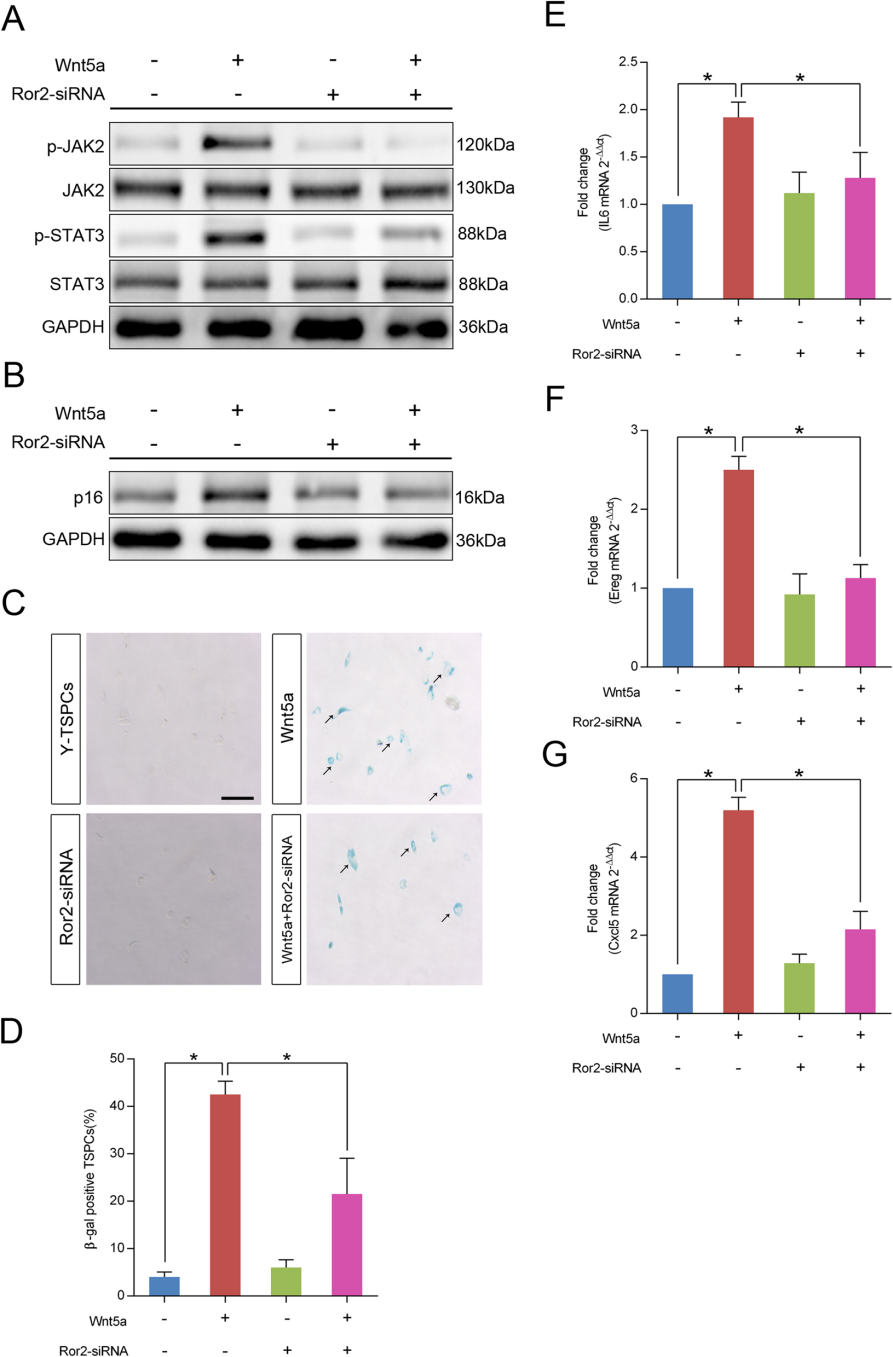
Wnt5a activates JAK–STAT signaling pathway through its co-receptor Ror2. **a** Young TSPCs were transfected with or without Ror2-siRNA and then stimulated with Wnt5a at 100 ng/ml for 16 h. The expressions of p-JAK2, JAK2, p-STAT3 and STAT3 were investigated by western blotting. **b** Western blotting for the p16^INK4A^ protein levels in young TSPCs upon Ror2-siRNA and/or Wnt5a (100 ng/ml, 16 h) treatment. **c**, **d** β-gal staining for the senescent cells in young TSPCs upon Ror2-siRNA and/or Wnt5a (100 ng/ml, 36 h) treatment. Scale bars: 200 μm. **e**–**g** qRT-PCR for IL6, Ereg and Cxcl5 mRNA levels in young, young TSPCs upon Ror2-siRNA and/or Wnt5a (100 ng/ml, 16 h) treatment. Values represent the mean ± SD. *p < 0.05, significantly different from the young or aged group

Wnt5a mediates noncanonical Wnt signaling which is transduced through frizzled (FZD) receptors and receptor tyrosine kinase-like orphan receptor (Ror) co-receptor [30, 31]. Here, we found that Wnt5a-induced increases in p-JAK2 and p-STAT3 levels were diminished by Ror2 knockdown in young TSPCs (Fig. 5a), which suggested that Wnt5a activates JAK–STAT signaling pathway depending on Ror2. Moreover, knockdown of Ror2 also suppressed Wnt5a-induced increases of p16^INK4A^ expression (Fig. 5b), as well as the number of β-gal-positive senescent cells (Fig. 5c, d). In addition, young TSPCs treated with Wnt5a showed increased levels of SASP genes, including IL6, Ereg and Cxcl5, and these increases were abolished when Wnt5a was administered together with Ror2-siRNA (Fig. 4e–g). Collectively, our results indicated that Ror2 acts as the functional receptor of Wnt5a in TSPCs senescence.

## Discussion

The association between tendon aging and TSPCs senescence is well recognized. In the present study, we demonstrated that Wnt5a expression is closely correlated with TSPCs senescence. Accordingly, we found that Wnt5a expression was significantly increased in aged TSPCs. Noncanonical Wnt5a inhibits Wnt/β-catenin signaling has been reported in various biological events [17, 18]. Consistent with previous studies, we also found that increased Wnt5a inhibited the activation of β-catenin and reduced canonical WNT target genes Axin2 and Lgr5 in aged TSPCs, which cause a switch from canonical to noncanonical Wnt signaling. In addition, Wnt5a knockdown attenuated cell senescence, resulting in decreased p16^INK4A^ expression and number of β-gal-positive cells in aged TSPCs. Senescence represents a state of permanent cell cycle arrest; as a cell cycle inhibitor, p16^INK4A^ has been proved to be an ideal marker for TSPCs senescence [32]. We also showed that Wnt5a knockdown promoted the cell cycle progression of aged TSPCs. Contrary to this, Wnt5a treatment promoted the young TSPCs senescence. In addition, Wnt5a knockdown also restored the age-related dysfunction of self-renewal, migration and tenogenic differentiation in TSPCs (Additional files 12, 13, 14: Figs. S8–S10). Collectively, Wnt5a is increased in aged TSPCs and activates noncanonical Wnt signaling, thereby contributing to TSPCs senescence.

Stem cells can propagate themselves through symmetric divisions, or divide asymmetrically to generate a daughter stem cell and a differentiating cell [33]. During asymmetric divisions, stem cells have the capacity to asymmetrically segregate damaged components. Such asymmetric division requires that a cell be polarized, and studies have suggested that loss of polarity is linked to stem cell aging [34]. In germ line stem cells, the age-related cell dysfunction correlates with loss of cell polarity [35]. In HSCs, elevated activity of Cdc42 correlates with a loss of cell polarity, and inhibition of CDC42 activity restores polarity in aged HSCs [25]. Tubulin has been used as a polarity marker for several stem cell populations [21, 35], and our study also suggested that tubulin could be an ideal marker in TSPCs showing distinct polarity phenotypes. In the present study, we reported for the first time that the polarity status of TSPCs changes upon aging, and we showed a reduction in cells with a polar distribution of tubulin in aged TSPCs. In addition, Wnt5a shRNA treatment restores polarity in aged TSPCs, which suggests a vital role of Wnt5a in the regulation of TSPCs polarity. We further propose that Wnt5a promotes the age-related dysfunction of TSPCs self-renewal and differentiation, at least in part, through the repression of cell polarity.

The SASP contains pro-inflammatory cytokines, chemokines, growth factor and matrix metalloproteinases, which constitutes a hallmark of senescent cells [36]. The SASP reinforces and spreads senescence in autocrine and paracrine manners, causing tissue microenvironment alteration and homeostasis disruption. Consistent with previous studies, our study also showed a robust pattern of SASP induction in aged TSPCs. Among these SASP factors, IL6 is a key proinflammatory cytokine playing critical roles in tendon repair [37]. EREG has been reported to play a potential role in tendon inflammation, and pro-inflammatory stimulation of tendon-like constructs causes a significant increase in the expression of EREG [38]. Our results raise the possibility that tendon aging may be driven by the SASP activation and the senescent TSPCs may serve as a major source of SASP factors. Moreover, we showed that inhibition of Wnt5a attenuated SASP expression in aged TSPCs, which suggested an essential and specific role of Wnt5a in regulating SASP in TSPCs. We speculated that increased Wnt5a will induce the accumulation of SASP factors in TSPCs and thus promote cellular senescence.

The JAK–STAT signaling is one of the important intracellular pathways, which is frequently activated in senescent stem cell [27, 39]. The JAK–STAT signaling pathway has a critical role in proliferation, migration and differentiation of stem cell. Our previous study has demonstrated that the JAK–STAT pathway was activated in aged TSPCs, which significantly contributes TSPCs senescence [32]. Here, we showed that overexpression of Wnt5a activates JAK–STAT pathways in young TSPCs and this appears to be a critical mechanism for Wnt5a-induced TSPCs senescence. Forced inhibition of JAK–STAT pathway through AG490 attenuated Wnt5a-induced TSPCs senescence. The results suggest that Wnt5a promotes senescence and age-related dysfunction of TSPCs might through JAK–STAT signaling pathway. In addition, studies have demonstrated that the JAK–STAT signaling pathway plays an important role in regulating cytokines and growth factors productions, which suggest that it may directly affect the SASP expression in TSPCs [39, 40]. Consistent with this possibility, we showed that inhibition of JAK–STAT pathway decreased the Wnt5a-induced SASP expressions in young TSPCs. Our study showed a novel role of JAK–STAT signaling pathway in Wnt5a-induced SASP expressions of TSPCs, and this could be another possible cause of TSPCs senescence.

The signal transduction of noncanonical Wnt activators requires distinct co-receptor Ror2 [41]. Ror2- and Wnt5a-deficient mice exhibit similar abnormalities during development, indicating that Wnt5a and Ror2 are physically and/or functionally linked [42]. Wnt5a-Ror2 signaling has been demonstrated to be involved in various physiological functions, such as differentiation, polarization and migration [43, 44]. In the present study, we found that knockdown of *Ror2* inhibited Wnt5a-induced TSPCs senescence, as well as the expressions of SASP genes. The results suggest that Ror2 is a key mediator of Wnt5a-induced senescence in TSPCs. In addition, we showed that knockdown of Ror2 inhibited the phosphorylation of JAK2 and STAT3, suggesting that the activation of the JAK–STAT signaling pathway by Wnt5a was mediated through Ror2. Collectively, our study further revealed the molecular mechanisms of increased TSPCs senescence by noncanonical Wnt5a and JAK–STAT signaling pathway.

## Conclusions

**Fig. 6:**
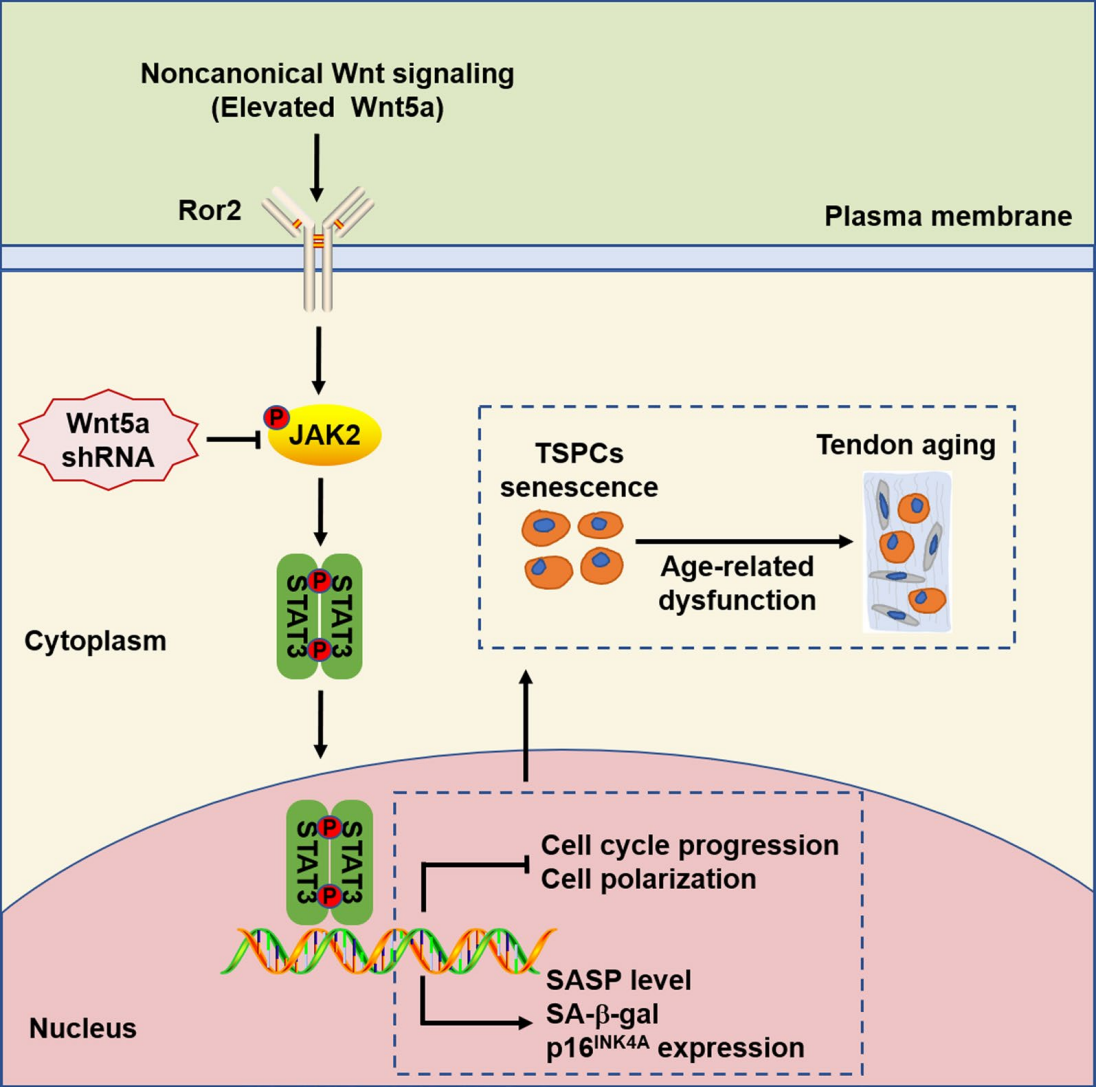
Schematic model describing the proposed mechanism of TSPCs aging. In aged TSPCs, increased noncanonical Wnt5a causes the activation of JAK–STAT signaling pathway, leading to cell senescence and impaired cell function, thereby promoting tendon aging

In summary, our study demonstrated that aberrant expression of noncanonical Wnt5a is an important contributor to cell senescence in TSPCs (Fig. 6). Functionally, inhibition of Wnt5a attenuated TSPCs senescence, age-related cell polarity and the SASP expression in aged TSPCs. We further found that Wnt5a modulates TSPCs senescence via a previously unknown mechanism to potentiate JAK–STAT signaling. Moreover, we showed that Ror2 acts as the functional receptor of Wnt5a in TSPCs senescence. Our findings suggest a novel essential mechanism involved in TSPCs senescence, which could be an ideal therapeutic agent for age-related tendon disorders.

## Supplementary Information

**Additional file 1**. Supplementary Materials and Methods.

**Additional file 2**. Table S1.

**Additional file 3**. Table S2.

**Additional file 4**. Supplementary Figure Legends.

**Additional file 5**.** Figure S1**. Histological analysis of young and aged tendons.

**Additional file 6**.** Figure S2**. Isolation and characterization of mouse TSPCs.

**Additional file 7**.** Figure S3**. Microarray and GO analysis of differentially expressed probe sets in young and aged TSPCs.

**Additional file 8**.** Figure S4**. Microarray and GO analysis of differentially expressed probe sets in aged and aged Wnt5a-knockdown TSPCs.

**Additional file 9**.** Figure S5**. Recombinant Wnt5a treatment promotes young TSPCs senescence.

**Additional file 10**.** Figure S6**. Gene expression analysis of aged and aged Wnt5a-knockdown TSPCs.

**Additional file 11**.** Figure S7**. Wnt5a is required for the activation of JAK-STAT signaling pathway in TSPCs.

**Additional file 12**.** Figure S8**. Wnt5a knockdown restores the self-renewal capacity of aged TSPCs.

**Additional file 13**.** Figure S9**. Wnt5a knockdown facilitates aged TSPCs migration.

**Additional file 14**.** Figure S10**. Wnt5a knockdown promotes tendon-related genes expressions of aged TSPCs.

## Data Availability

All data generated or analyzed during this study are included in this published article [and its supplementary information files].

## Abbreviations

TSPCs: Tendon stem/progenitor cells
Wnt5a: Wingless-type MMTV integration site family, member 5A
JAK2: Janus kinase 2
STAT3: Signal transducer and activator of transcription 3
Ror2: Receptor tyrosine kinase-like orphan receptor 2
SASP: Senescence-associated secretory phenotype
shRNA: Short-hairpin RNA
β-gal: β-Galactosidase
HDFs: Human diploid fibroblasts
HSCs: Hematopoietic stem cells
GO: Gene ontology
GSEA: Gene set enrichment analysis
