# Supplementary material for: Noncanonical Wnt5a signaling regulates tendon stem/progenitor cells senescence
Source: Stem Cell Res Ther. 2021 Oct 18;12:544. doi: 10.1186/s13287-021-02605-1 (PMC8521898; doi:10.1186/s13287-021-02605-1)
Supplement: Supplementary file 1 — Additional file 1. Supplementary Materials and Methods. [file 13287_2021_2605_MOESM1_ESM.docx]

**Supplementary Materials and Methods**

**Histochemical analysis**

Achilles tendons were fixed in 4% paraformaldehyde for 24 h, then dehydrated, embedded in paraffin, 5-μm sections were obtained and examined. Sections were stained with hematoxylin-eosin, Alizarin red or Safranin O (all from Servicebio). Images were captured by an Olympus CKX53 inverted phase-contrast microscope.

**Flow cytometry assay**

TSPCs (1×10^6^) at P3 were incubated with 0.25 μg of APC-labeled anti-CD73, PE-labeled anti-CD105, FITC-labeled anti-CD90.2 or PE/Cy5-labeled anti-CD34 and isotype-matched IgGs (all from Biolegend) for 30min at 4°C. After washing in PBS for three times, the stained cells were re-suspended in 400 μL PBS and subjected to FACS analysis (BD Biosciences).

**Osteogenic differentiation assays**

For osteogenic differentiation, TSPCs (2×10^4^ cells/cm^2^) were plated into a gelatin-coated 6-well plate and cultured in complete culture medium until the cells reached 70% confluence. Then the cells were cultured in osteogenic induction medium (Cyagen Biosciences) for 21 days, the medium was changed every 3 days. For Alizarin red staining, the cells were fixed with 4% paraformaldehyde for 30 minutes and stained with Alizarin red (Cyagen Biosciences) for 5 min.

**Adipogenic differentiation assays**

For adipogenic differentiation, TSPCs (2×10^4^ cells/cm^2^) were plated into a 6-well plate and cultured in complete culture medium until the cells reached 100% confluence. Then the cells were cultured in adipogenic induction medium (Cyagen Biosciences) for 24 days according to the manufacturer’s instruction. For Oil Red O staining, the cells were fixed with 4% paraformaldehyde for 30 minutes and stained with Oil Red O (Cyagen Biosciences) for 30 min.

**Chondrogenic differentiation assays**

For chondrogenic differentiation, a pellet culture system was used. About 4 × 10^5^ cells were pelleted into a micro mass by centrifugation at 150 g for 5 min in a 15-mL centrifuge tube and cultured in chondrogenic induction medium (Cyagen Biosciences) for 28 days according to the manufacturer’s instruction. For Alcian blue staining, the section of pellet was deparaffinized, dehydrated, and then stained with Alcian blue (Cyagen Biosciences) for 30 min.

**Colony forming unit (CFU) assays**

For the CFU assay, 5, 10 and 20 cells/cm^2^ of TSPCs were plated in 6-well plates respectively for 14 days in complete media. The cells were stained with 0.5% crystal violet for counting the number of cell colonies.

**CCK-8 assay**

The Cell Counting Kit-8 (CCK-8, Keygen biotech) assay was used to measure cell proliferation. Cells were plated into 96-well culture plates at an optimal density of 10000 cells/well in 100 μL complete culture medium. The cells were observed under a microscope and the CCK-8 assay was performed at 0 h, 24 h, 48 h and 72 h. Then the 10ul CCK8 solution was added to each well and incubated for 4 h at 37 °C, the absorbance of each well was read by the microplate reader at 450 nm.

**Population doubling time (PDT) assay**

Population doubling time (PDT) assay was calculated from the formula log_2_ [Nc/N0], where N0 refers to the total cell number during seeding, and Nc is the total cell number at confluence.

**TSPCs migration assay**

TSPCs were plated on 6-well plates and grown to confluence. Then the medium was removed, and the monolayer was scratched with a sterile plastic pipette tip. The TSPCs were washed with PBS and incubated for 18 h before being imaging under an inverted microscope. The initial scratch length and scratch bridging time were measured and used for calculation of cell velocity. Images were captured by an Olympus CKX53 inverted phase-contrast microscope.

**Investigation of actin dynamics**

For actin dynamics analysis, the young, aged and aged Wnt5a-knockdown TSPCs were plated on 6-well plates and incubated for 48 h. Then cells were treated with 0.4 μM Latrunculin A (Sigma-Aldrich) in a time-dependent manner (0, 15, 30 and 60 min). Cells were fixed in 4% paraformaldehyde and permeabilized with 0.1% Triton X-100, then the cells were stained with Alexa Flour 546 phalloidin (Thermo Scientific). Immunofluorescence was visualized with a Nikon Ts2R fluorescence microscope.
