# Supplementary material for: Noncanonical Wnt5a signaling regulates tendon stem/progenitor cells senescence
Source: Stem Cell Res Ther. 2021 Oct 18;12:544. doi: 10.1186/s13287-021-02605-1 (PMC8521898; doi:10.1186/s13287-021-02605-1)
Supplement: Supplementary file 2 — Additional file 2. Table S1. [file 13287_2021_2605_MOESM2_ESM.docx]

| Table s1. Biological functions and genes affected after transfection | |
| --- | --- |
| Categories | Genes |
| Cytokine activity | Gdf15, Il16, Ccl9, Lif, Inhbb, Inhba, Ccl2, Ccl7, Gdf6, Cxcl1, Il6, Crlf1, Cxcl12, Cx3cl1, Tnfsf11, Csf3, Il33, Ccl5, Nampt, Cxcl10, Cxcl5, Cxcl14, Bmp4, Ccl28, C1qtnf4, Il11, Lefty1, Clcf1, Cmtm7, Kitl |
| Chemokine activity | Ccl5, Cxcl10, Ccl9, Cxcl14, Cxcl5, Ccl2, Ccl7, Ccl28, Cxcl1, Cxcl12, Cx3cl1 |
| Growth factor activity | Gmfg, Gdf15, Cdnf, Fgf21, Figf, Cxcl1, Ctgf, Gfer, Nov, Pdgfb, Hdgf, Igf1, Bmp4, Thbs4, Lefty1, Ereg, Prl2c3, Kitl, Prl2c2, Efemp1, Inhbb, Hgf, Ptn, Lif, Inhba, Gdf6, Fgf14, Il6, Cxcl12, Pdgfd, Tgfa, Igf2, Csf3, Ngf, Btc, Fgf13, Fgf7, Ogn, Fgf18, Mdk, Il11, Fgf10 |
| Cytokine receptor binding | Gdf15, Ccl2, Ccl7, Fkbp1a, Figf, Pik3r1, Tlr5, Cxcl1, Nes, Cx3cl1, Eda, Angpt1, Rasl11b, Ccl5, Il1rap, Cxcl10, Cxcl14, Cxcl5, Efna5, Traf6, Itch, Bmp4, Ccl28, Il6ra, Dab2ip, Lefty1, Il6st, Pdcl3, Lifr, Fadd, Timm50, Kitl, Traf3, Ccl9, Siva1, Lif, Inhbb, Traf4, Inhba, Gdf6, Nup85, Il6, Crlf1, Traf1, Cxcl12, Tnfsf11, Csf3, Ngf, Tgfbr3, Smad7, Il11, Clcf1, Cnih4 |
| Cytokine receptor activity | Osmr, Il1rap, Il1r1, Gfra2, Ackr3, Il20ra, Il6ra, Il17rd, Ccr10, Il6st, Lifr, F3, Il18r1, Ghr |
| Growth factor receptor binding | Flrt2, Il1r1, Efemp1, Fgf21, Rnf126, Figf, Pik3r1, Tlr5, Il6, Pdgfd, Tgfa, Angpt1, App, Csf3, Pdgfb, Il1rap, Btc, Fgf7, Il6ra, Dab2ip, Fgf18, Il11, Il6st, Pdcl3, Ereg, Pten, Fgf10, Pdgfra, Lingo1, Flrt1 Timm50 |
