# Supplementary material for: Noncanonical Wnt5a signaling regulates tendon stem/progenitor cells senescence
Source: Stem Cell Res Ther. 2021 Oct 18;12:544. doi: 10.1186/s13287-021-02605-1 (PMC8521898; doi:10.1186/s13287-021-02605-1)
Supplement: Supplementary file 3 — Additional file 3. Table S2. [file 13287_2021_2605_MOESM3_ESM.docx]

TABLE S2. Primer sequences for qRT-PCR.

| Target gene | Accession No. | Forward | Reverse | | Amplicon (bp) |
| --- | --- | --- | --- | --- | --- |
| Wnt5a | NM_001256224.2 | 5′-GCTTCGCCAAGGAGTTCGTG-3′ | 5′-TGTATACTGTCCTACGGCCTGCT-3′ | 123 | |
| Axin2 | NM_015732.4 | 5′-CATAGTGCCCAAAGCATAAGAAAG-3′ | 5′-GGTCCTGGGTAAATGGGTGAG-3′ | 142 | |
| Lgr5 | NM_010195.2 | 5′-ACTACGCCTTTGGAAACCTCTC-3′ | 5′-AGAGTGTCTTGATTGCAGTGGG-3′ | 162 | |
| p16^INK4A^ | NM_009877.2 | 5′-GCTTCTCACCTCGCTTGTCAC-3′ | 5′-AGAAAACCCTCTCTTGGAGTGG-3′ | 128 | |
| IL6 | NM_031168.2 | 5′-TTCTTGGGACTGATGCTGGTG-3′ | 5′-GCCATTGCACAACTCTTTTCTC-3′ | 177 | |
| IL16 | NM_001360087.1 | 5′-ACCAAAGGACACGCAGCAACT-3′ | 5′-CTTGGCCCTTCATCAGCACTA-3′ | 267 | |
| Cxcl1 | NM_008176.3 | 5′-CACCCAAACCGAAGTCATAGC-3′ | 5′-GGGGACACCTTTTAGCATCTTT-3′ | 109 | |
| Cxcl5 | NM_009141.3 | 5′-GTGTTTGCTTAACCGTAACTCCA-3′ | 5′-GTGCATTCCGCTTAGCTTTCT-3′ | 210 | |
| Cxcl12 | NM_001012477.2 | 5′-AGTCAGCCTGAGCTACCGATG-3′ | 5′-TTCTTCAGCCGTGCAACAATC-3′ | 126 | |
| Ereg | NM_007950.2 | 5′-CTCCCTGCCTCTTGGGTCTT-3′ | 5′-TTCCATCTGAACTAAGGCGGTAC-3′ | 135 | |
| Tnfsf11 | NM_011613.3 | 5′-CCATCGGGTTCCCATAAAGTCA-3′ | 5′-CAGTTTTTCGTGCTCCCTCCTT-3′ | 266 | |
| Ccl2 | NM_011333.3 | 5′-GCAGGTCCCTGTCATGCTTCT-3′ | 5′-TGTCTGGACCCATTCCTTCTTG-3′ | 253 | |
| Tnmd | NM_022322.2 | 5′-ACCAGACAAGCAAGCGAGGAA-3′ | 5′-CAAGGCATGATGACACGACAGAT-3′ | 215 | |
| Col1A1 | NM_007742.4 | 5′-GAGAGGTGAACAAGGTCCCG-3′ | 5′-AAACCTCTCTCGCCTCTTGC-3′ | 153 | |
| Nestin | NM_016701.3 | 5′-CACACCTCAAGATGTCCCTTAGTC-3′ | 5′-GGGTCAGGAAAGCCAAGAGA-3′ | 121 | |
| Scx | NM_198885.3 | 5′-AGAACACCCAGCCCAAACAG-3′ | 5′-ATCGCCGTCTTTCTGTCACG-3 | 85 | |
| Bgn | NM_007542.5 | 5′-GGATGATTGAGAATGGGAGCC-3′ | 5′-TCAGTAACGCAGCGGAAGG-3′ | 274 | |
| β-actin | NM_007393.3 | 5′-GTGACGTTGACATCCGTAAAGA-3′ | 5′-GTAACAGTCCGCCTAGAAGCAC-3′ | 287 | |
