# Supplementary material for: Noncanonical Wnt5a signaling regulates tendon stem/progenitor cells senescence
Source: Stem Cell Res Ther. 2021 Oct 18;12:544. doi: 10.1186/s13287-021-02605-1 (PMC8521898; doi:10.1186/s13287-021-02605-1)
Supplement: Supplementary file 4 — Additional file 4. Supplementary Figure Legends. [file 13287_2021_2605_MOESM4_ESM.docx]

**Supplementary Figure Legends**

Fig. S1 Histological analysis of young (2-month-old) and aged tendons (20-month-old). (A, B) Longitudinal sections of achilles tendons were stained with hematoxylin-eosin (H&E). The aged tendons showed irregular collagen fibers and loss of collagen stainability. Scale bar: 20 μm. (C, D) Alizarin red staining showed a significant calcification site in the aged tendons. Scale bar: 20 μm. (E, F) Proteoglycan content was assessed with Safranin O staining in young and aged tendons, the result revealed a reduction of proteoglycan content in aged tendon. Scale bar: 20 μm.

Fig. S2 Isolation and characterization of mouse TSPCs. (A) Colony forming unit (CFU) assay was performed at three densities, crystal violet stained colonies at day 14. Scale bars: 200μm. (B) Flow cytometry analysis of the expression of cell surface markers related to mesenchymal stem cells markers (CD73 and CD105), fibroblast marker (CD90.2) and hematopoietic stem cell marker (CD34) on TSPCs. (C) Adipogenesis potential of TSPCs, the lipid vacuoles were stained with Oil Red O at day 24. Scale bars: 200μm. (D) Osteogenesis potential of TSPCs was detected by Alizarin red staining at day 21. Scale bars: 200μm. (E) Chondrogenesis was induced in pellet culture, the section of pellet was stained with Alcian blue at day 28. Scale bars: 200μm.

Fig. S3 Microarray and gene ontology (GO) analysis of differentially expressed probe sets in young and aged TSPCs. (A) Heatmap showed genes that were differentially expressed between young and aged TSPCs. (B-D) GO terms with the most significant p values for molecular function, biological processes and cellular component.

Fig. S4 Microarray and GO analysis of differentially expressed probe sets in aged and aged Wnt5a-knockdown TSPCs. (A, B) The transfection efficiency of Wnt5a shRNA was investigated by western blotting and qRT-PCR. (C) Heatmap showed genes that were differentially expressed between aged and aged Wnt5a-knockdown TSPCs. (D-F) GO terms with the most significant p values for biological processes, molecular function and cellular component. Values represent the mean ± SD. *P < 0.05, significantly different from the young or aged group.

Fig. S5 Recombinant Wnt5a treatment promotes young TSPCs senescence. (A) Western blotting for the p16^INK4A^ protein level in young TSPCs and young TSPCs treated with Wnt5a (100 ng/ml, 16h). (B) β-gal staining for the senescent cells in young TSPCs and young TSPCs treated with Wnt5a (100 ng/ml, 24h). Scale bars: 100μm. (C) Quantitative analysis of β-gal positive TSPCs. (D) Flow cytometry analysis for the cell cycle distribution of TSPCs. Values represent the mean ± SD. *P < 0.05, significantly different from the young group.

Fig. S6 Gene expression analysis of aged and aged Wnt5a-knockdown TSPCs. (A) The GSEA KEGG analysis revealed the TOP 10 significant enriched signaling pathways in aged TSPCs.

Fig. S7 Wnt5a is required for the activation of JAK-STAT signaling pathway in TSPCs. (A) Young treated with Wnt5a (100ng/ml) and/or AG490 (10 Μm) for 24h. The protein levels of p-JAK2, JAK2, p-STAT3 and STAT3 were investigated by western blotting. (B) Western blotting for the p16^INK4A^ protein levels in young TSPCs upon Wnt5a (100 ng/ml) and/or AG490 (10 μM) treatment for 24h. (C) β-gal staining for the senescent cells in young TSPCs upon Wnt5a (100 ng/ml) and/or AG490 (10 μM) treatment for 24h. Scale bars: 100μm. (D) Quantitative analysis of β-gal positive TSPCs. (E-G) qRT-PCR for IL6, Ereg, and Cxcl5 mRNA levels in young, young TSPCs upon upon Wnt5a (100 ng/ml) and/or AG490 (10 μM) treatment for 24h. Values represent the mean ± SD. *P < 0.05, significantly different from the young TSPCs or young TSPCs treated with Wnt5a.

Fig. S8 Wnt5a knockdown restores the self-renewal capacity of aged TSPCs. (A) Colony forming unit (CFU) assay was performed at three densities, crystal violet stained colonies at day 14. (B, C) Proliferation rate of TSPCs was measured by population doubling time CCK-8 assay and (PDT) assay. Values represent the mean ± SD. *P < 0.05, significantly different from the young or aged group.

Fig. S9 Wnt5a knockdown facilitates aged TSPCs migration. (A) Scratch assay of young, aged and aged Wnt5a-knockdown TSPCs. Scale bar: 200 μm. (B, C) Quantification of scratch bridging time and cell velocity. (D) Phalloidin labeling at different time points in TSPCs after latrunculin A treatment. Wnt5a knockdown reduced the F-actin content in aged TSPCs, which indicated the improved actin turnover of aged TSPCs. Scale bar: 50 μm. The F-actin content at the beginning (0 min) was set to 100%. Values represent the mean ± SD. *P < 0.05, significantly different from the young or aged group.

Fig. S10 Wnt5a knockdown promotes tendon-related genes expressions of aged TSPCs. (A-E) Relative mRNA levels of representative tendon-related genes (Tnmd, Col1A1 Nestin, Scx and Bgn) in young, aged and aged Wnt5a-knockdown TSPCs were investigated by qRT-PCR. Values represent the mean ± SD. *P < 0.05, significantly different from the young or aged group.
