# Supplementary figures and images for: Noncanonical Wnt5a signaling regulates tendon stem/progenitor cells senescence
Source: Stem Cell Res Ther. 2021 Oct 18;12:544. doi: 10.1186/s13287-021-02605-1 (PMC8521898; doi:10.1186/s13287-021-02605-1)

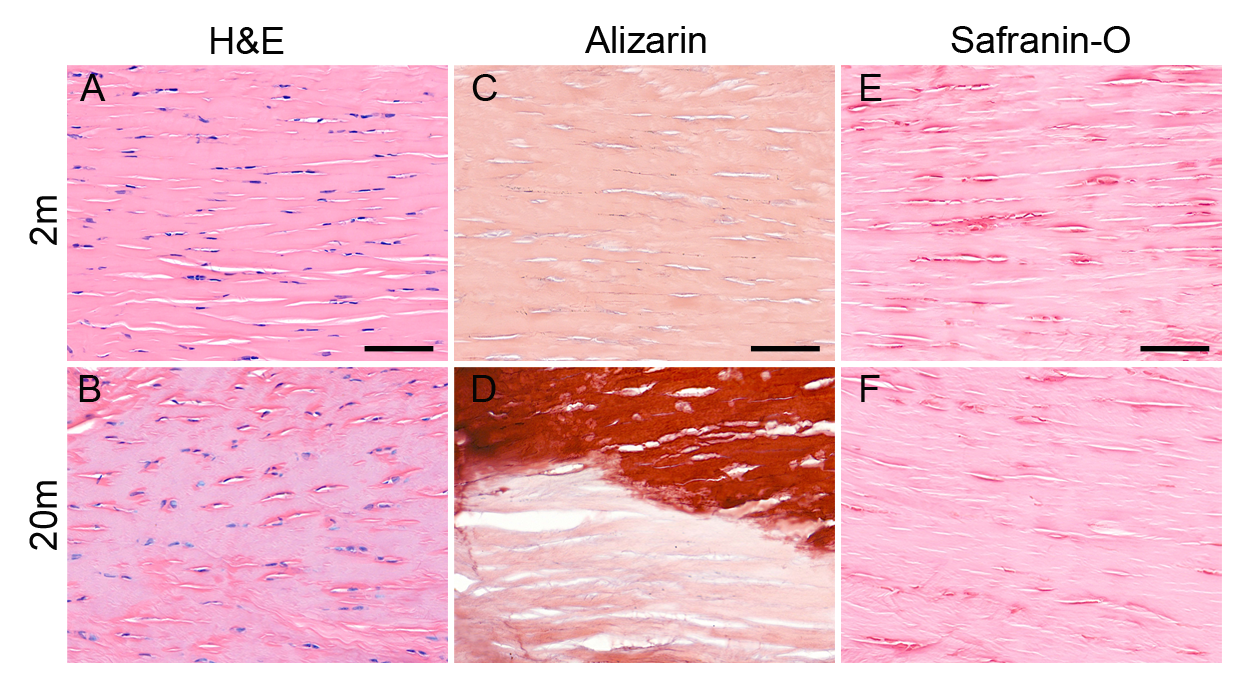

Supplement: Supplementary file 5 — Additional file 5. Figure S1. Histological analysis of young and aged tendons. [file 13287_2021_2605_MOESM5_ESM.tif]

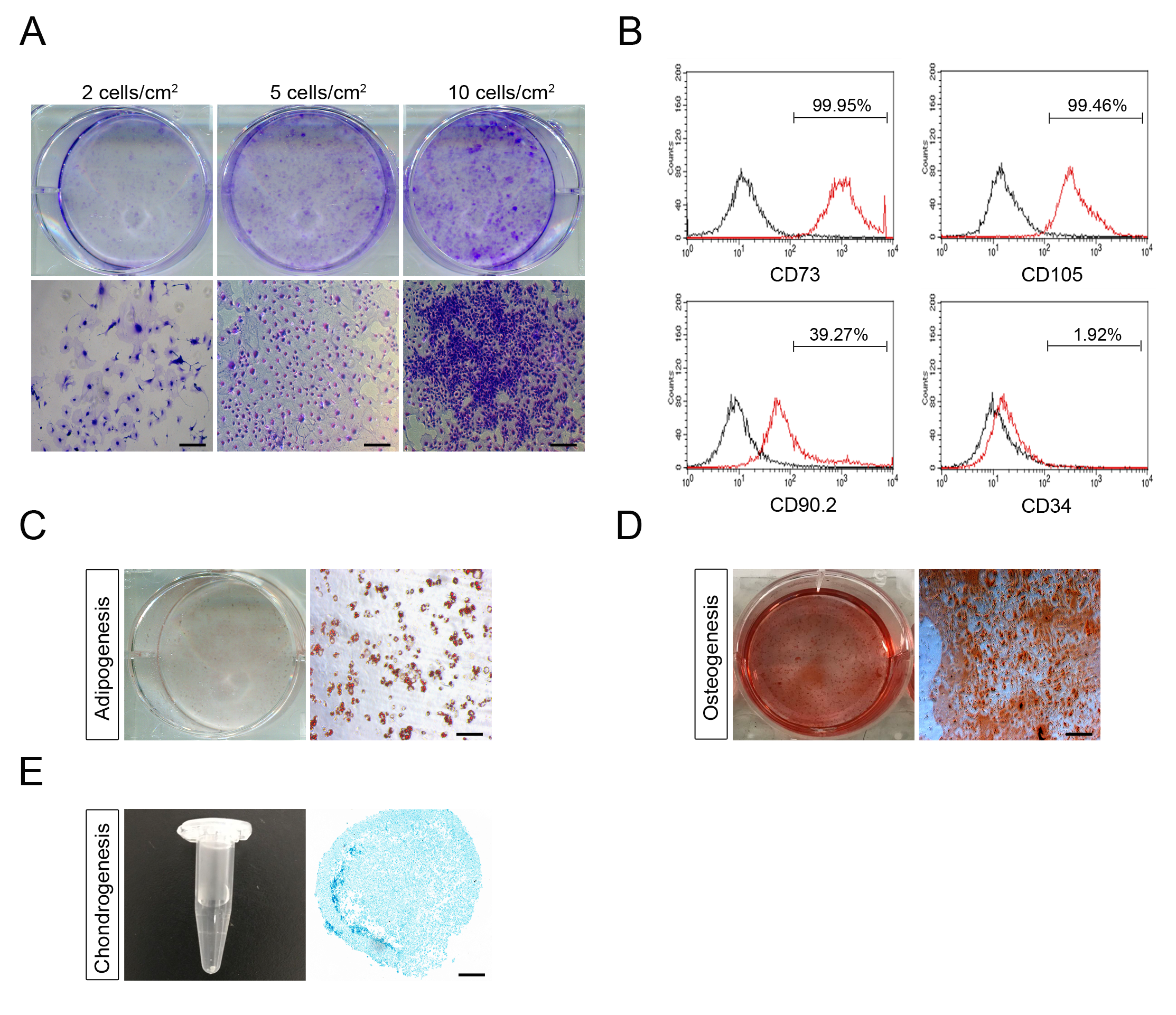

Supplement: Supplementary file 6 — Additional file 6. Figure S2. Isolation and characterization of mouse TSPCs. [file 13287_2021_2605_MOESM6_ESM.tif]

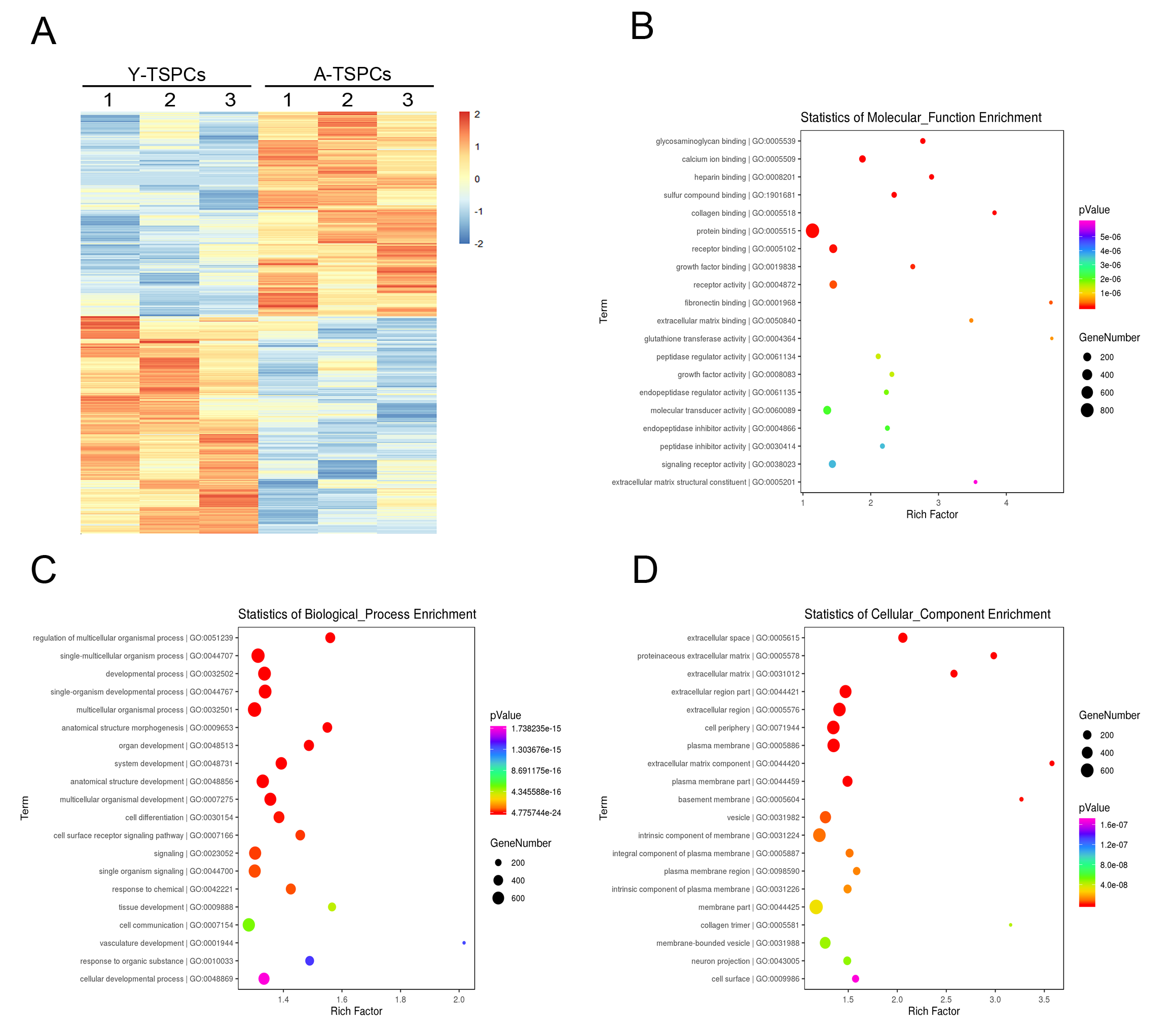

Supplement: Supplementary file 7 — Additional file 7. Figure S3. Microarray and GO analysis of differentially expressed probe sets in young and aged TSPCs. [file 13287_2021_2605_MOESM7_ESM.tif]

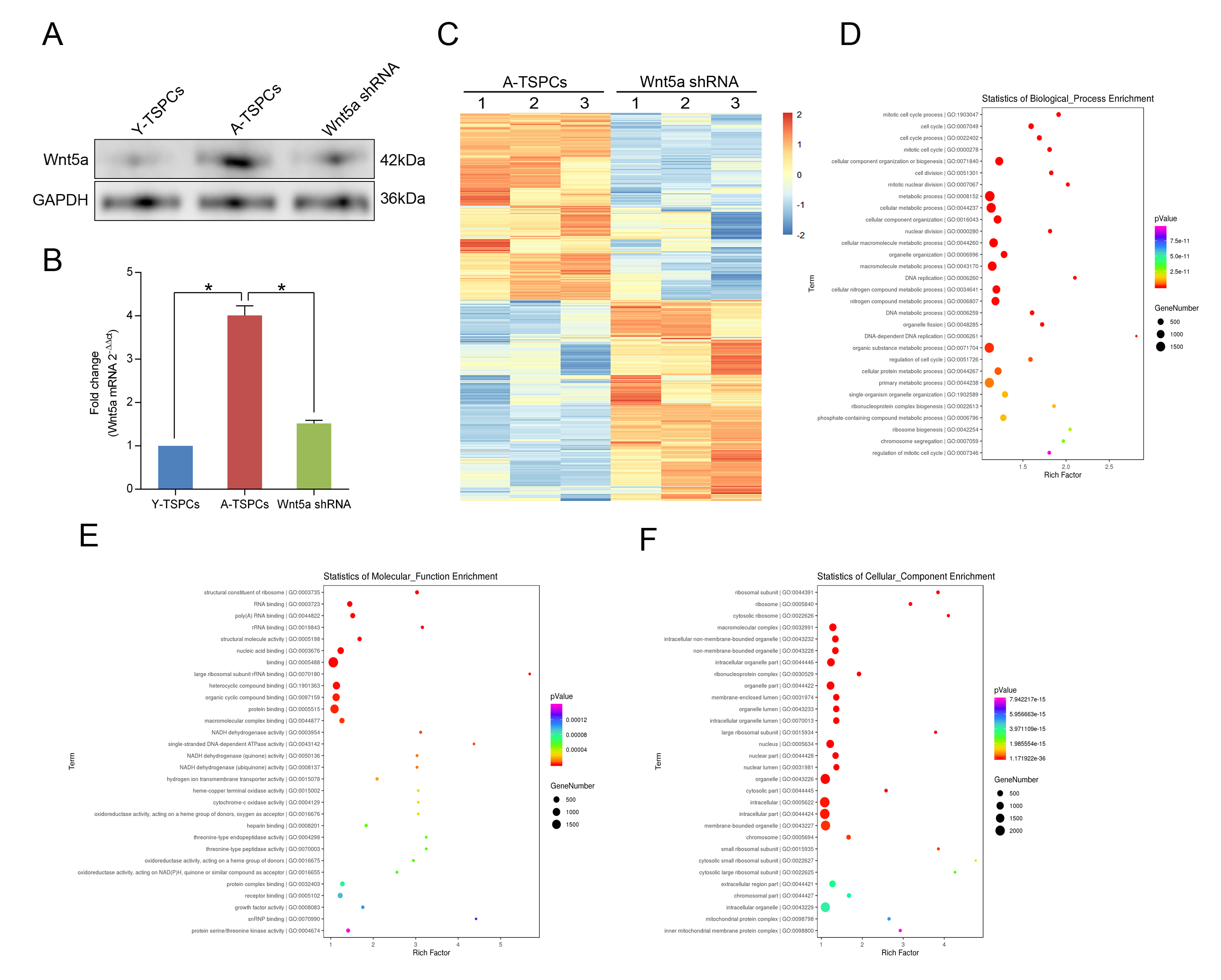

Supplement: Supplementary file 8 — Additional file 8. Figure S4. Microarray and GO analysis of differentially expressed probe sets in aged and aged Wnt5a-knockdown TSPCs. [file 13287_2021_2605_MOESM8_ESM.tif]

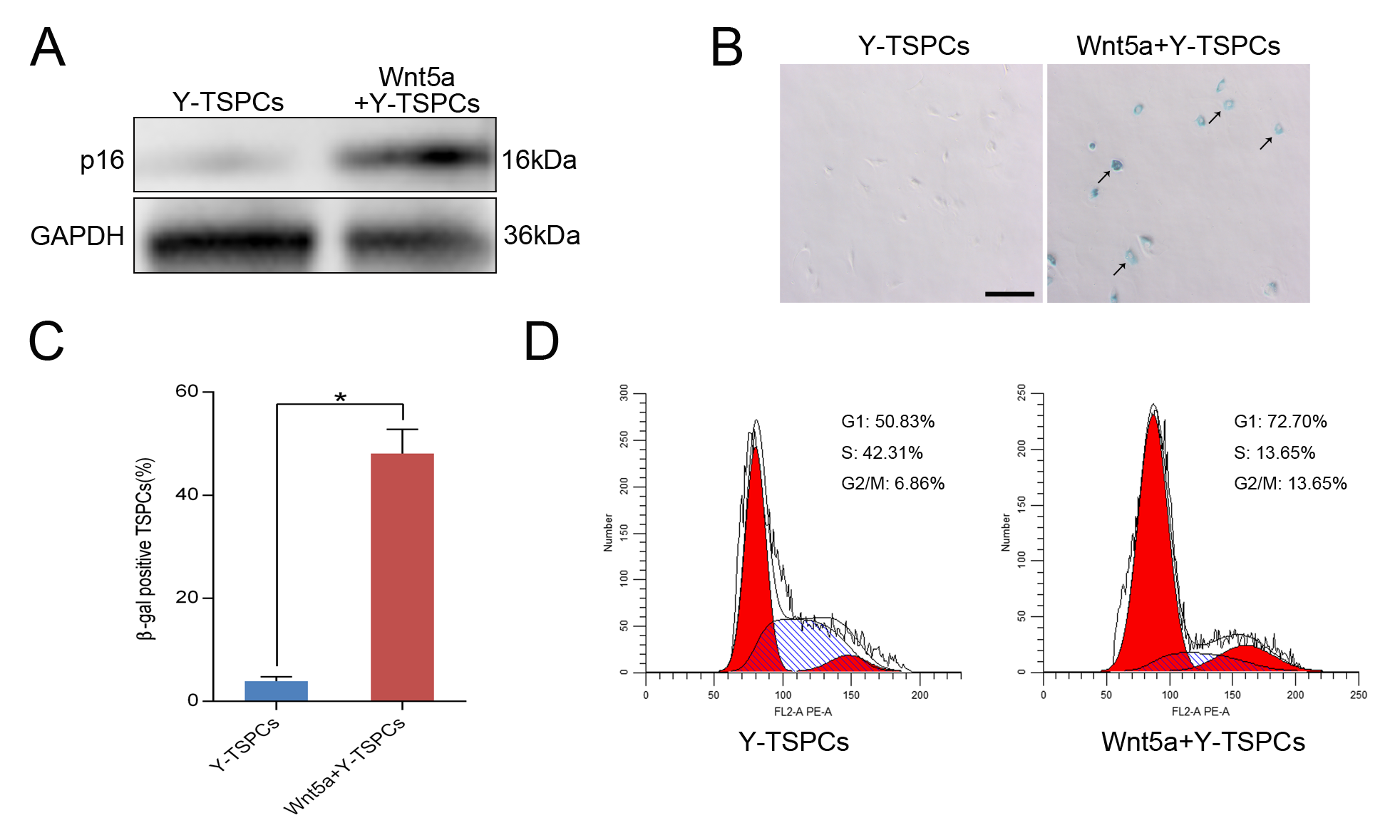

Supplement: Supplementary file 9 — Additional file 9. Figure S5. Recombinant Wnt5a treatment promotes young TSPCs senescence. [file 13287_2021_2605_MOESM9_ESM.tif]

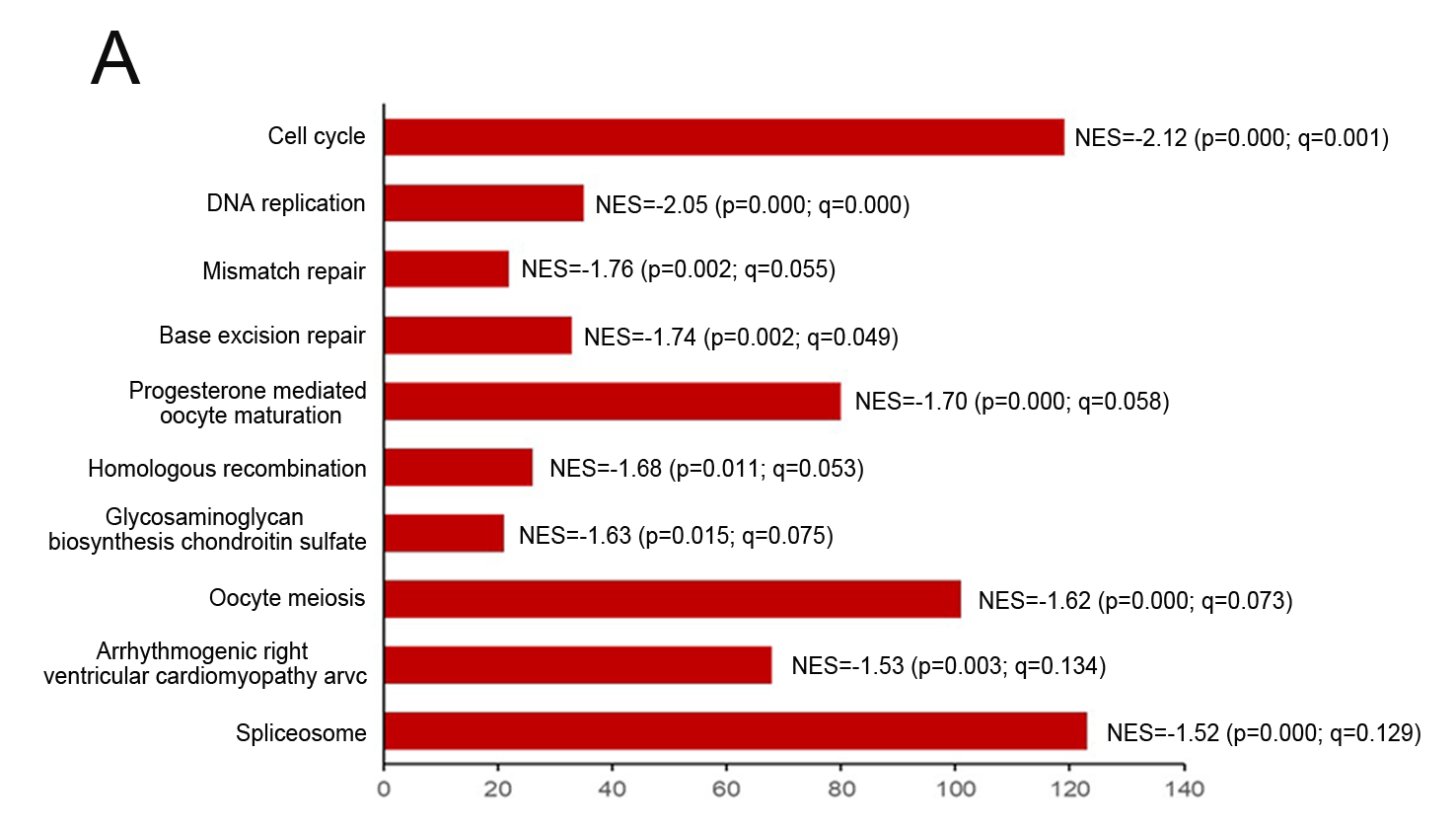

Supplement: Supplementary file 10 — Additional file 10. Figure S6. Gene expression analysis of aged and aged Wnt5a-knockdown TSPCs. [file 13287_2021_2605_MOESM10_ESM.tif]

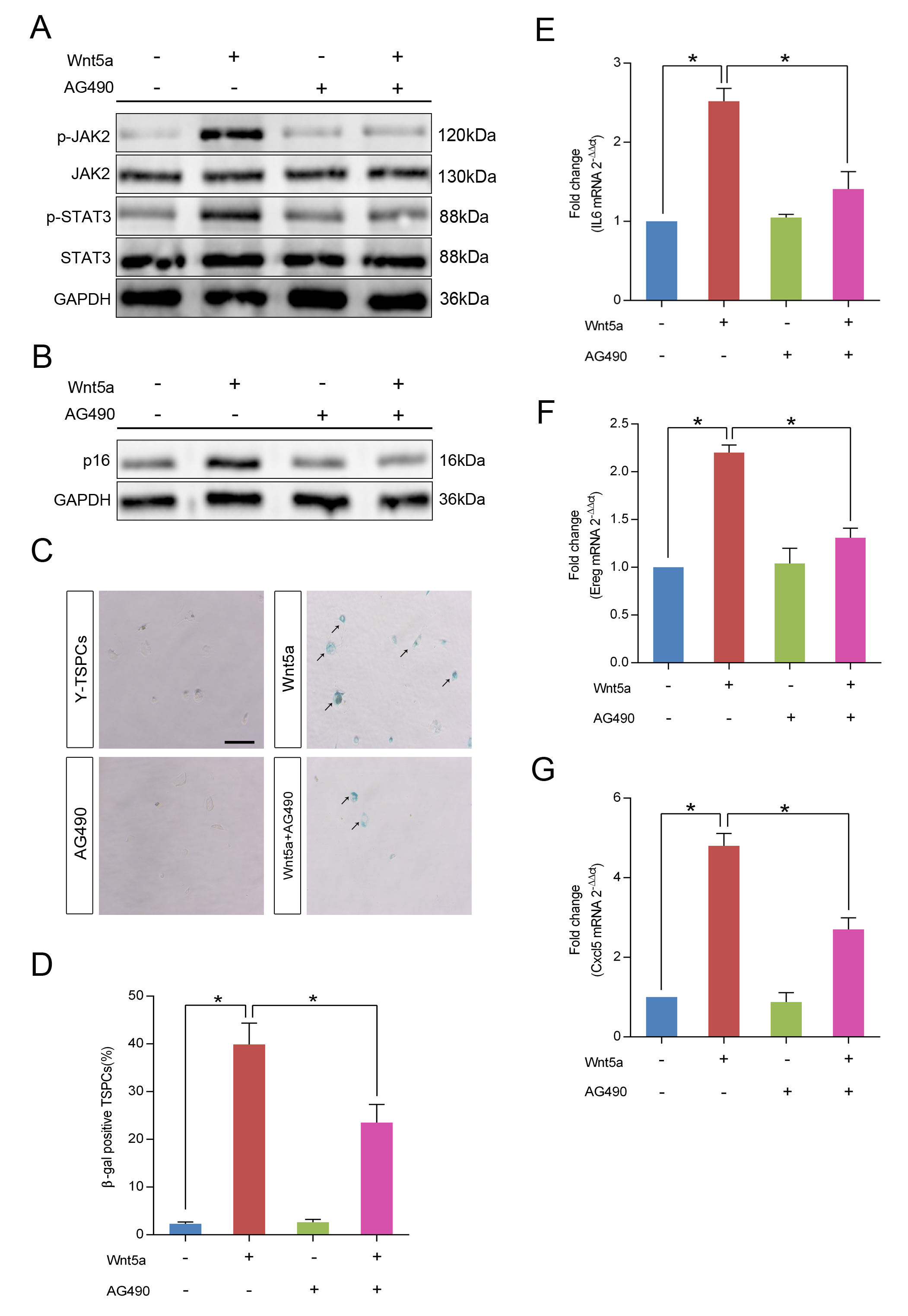

Supplement: Supplementary file 11 — Additional file 11. Figure S7. Wnt5a is required for the activation of JAK-STAT signaling pathway in TSPCs. [file 13287_2021_2605_MOESM11_ESM.tif]

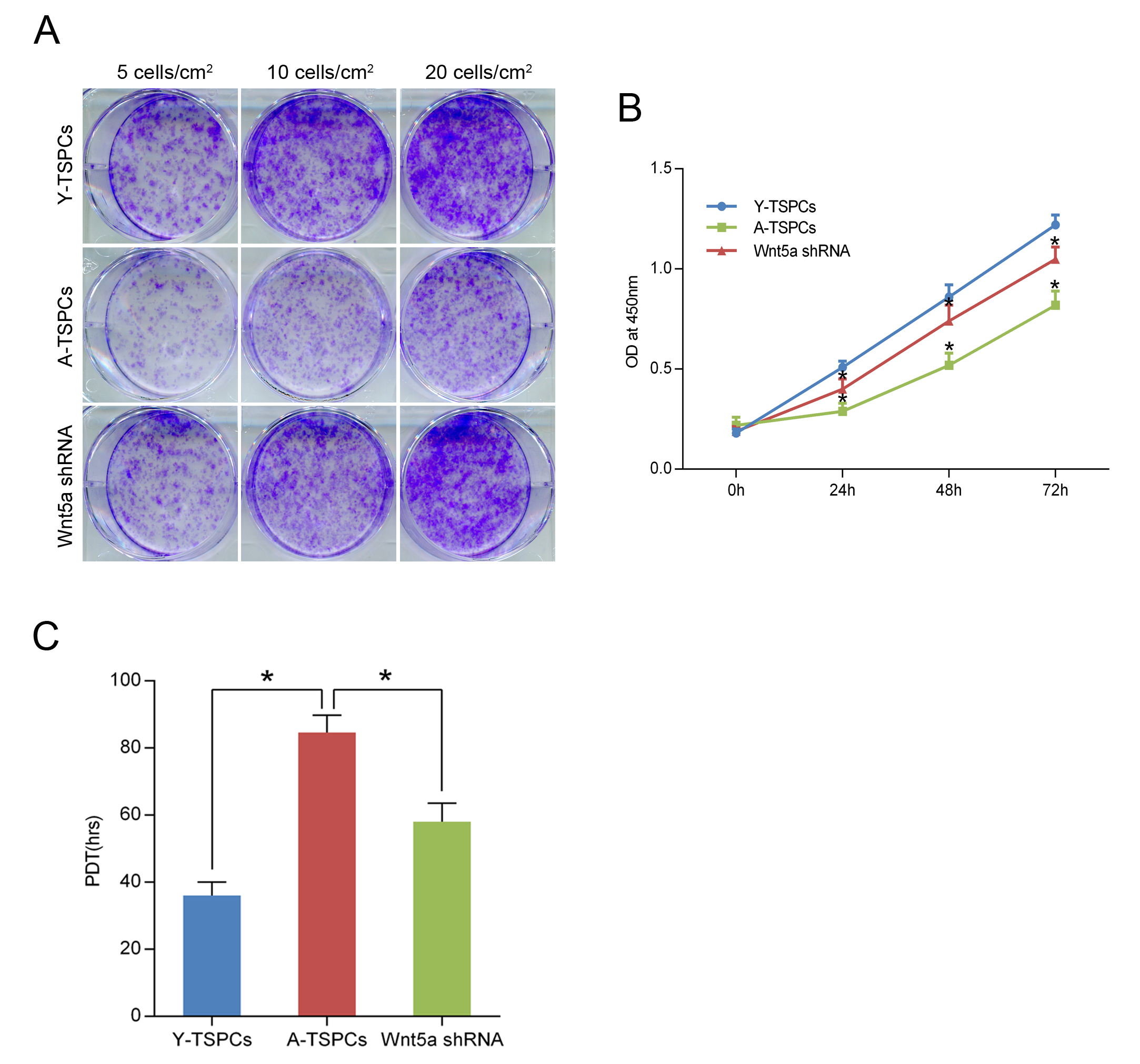

Supplement: Supplementary file 12 — Additional file 12. Figure S8. Wnt5a knockdown restores the self-renewal capacity of aged TSPCs. [file 13287_2021_2605_MOESM12_ESM.tif]

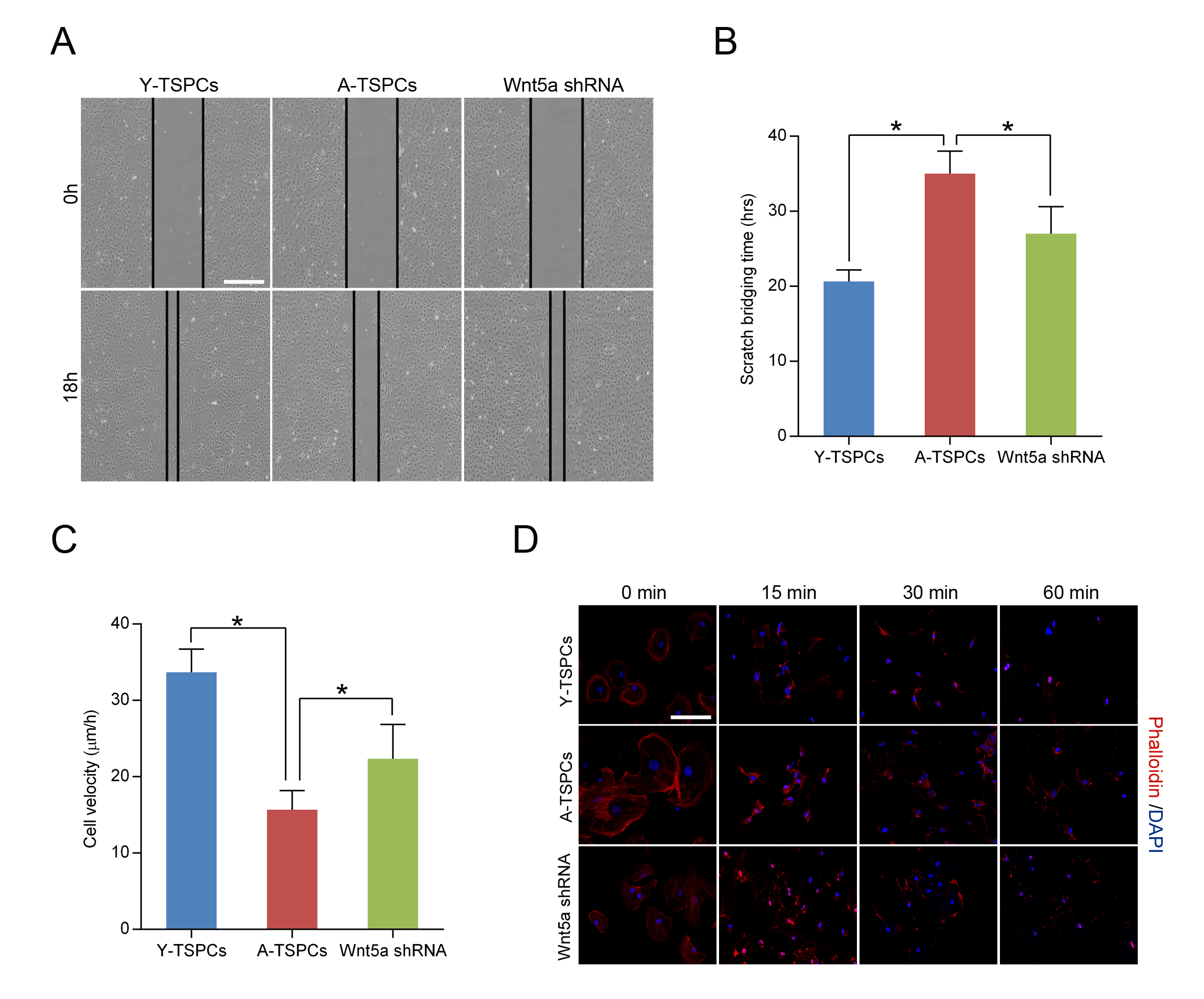

Supplement: Supplementary file 13 — Additional file 13. Figure S9. Wnt5a knockdown facilitates aged TSPCs migration. [file 13287_2021_2605_MOESM13_ESM.tif]

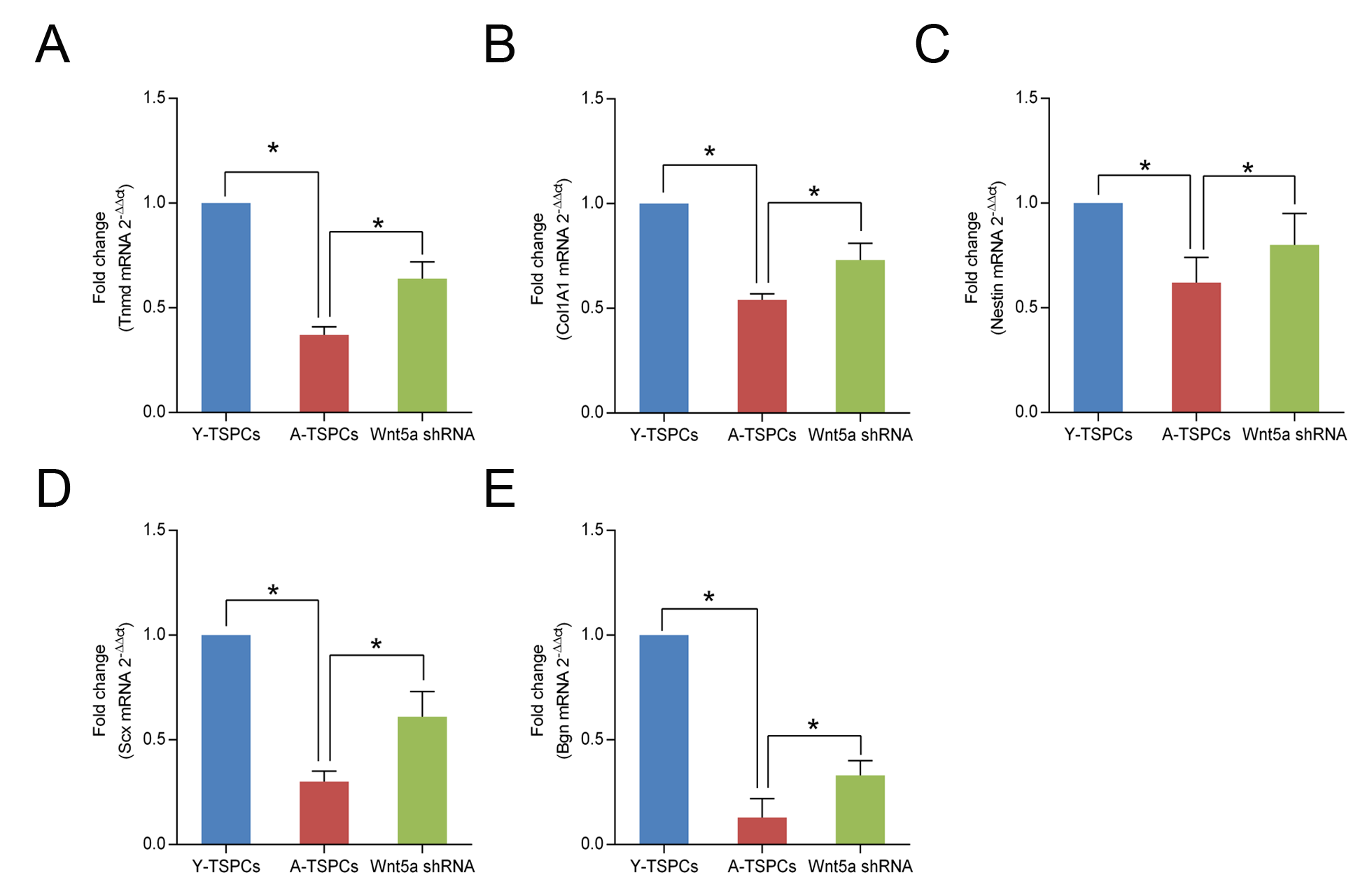

Supplement: Supplementary file 14 — Additional file 14. Figure S10. Wnt5a knockdown promotes tendon-related genes expressions of aged TSPCs. [file 13287_2021_2605_MOESM14_ESM.tif]
